# Supplementary figures and images for: Music compensates for altered gene expression in age-related cognitive disorders
Source: Sci Rep. 2023 Dec 2;13:21259. doi: 10.1038/s41598-023-48094-5 (PMC10692168; doi:10.1038/s41598-023-48094-5)

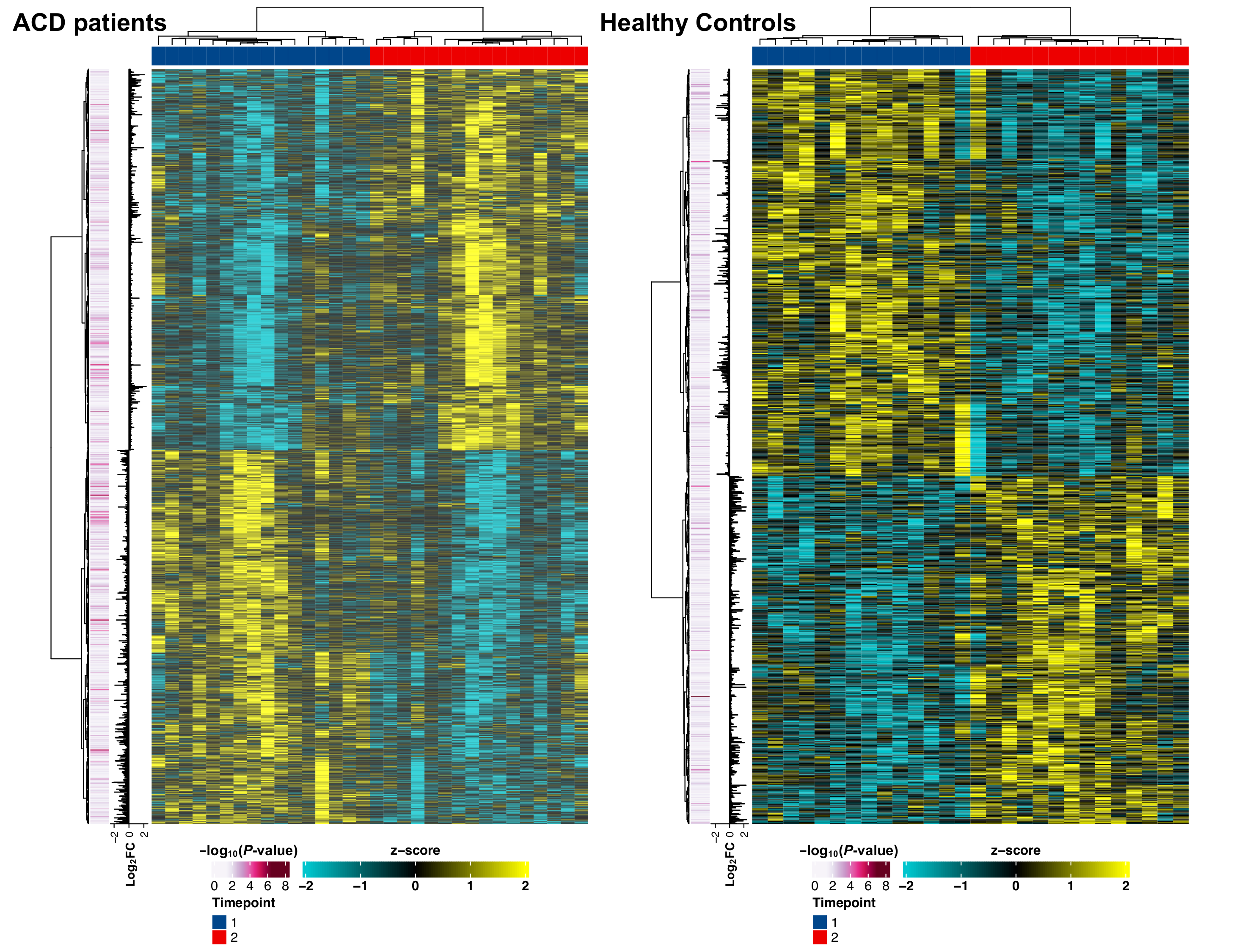

Supplement: Supplementary file 1 — Supplementary Figure S1. [file 41598_2023_48094_MOESM1_ESM.tif]

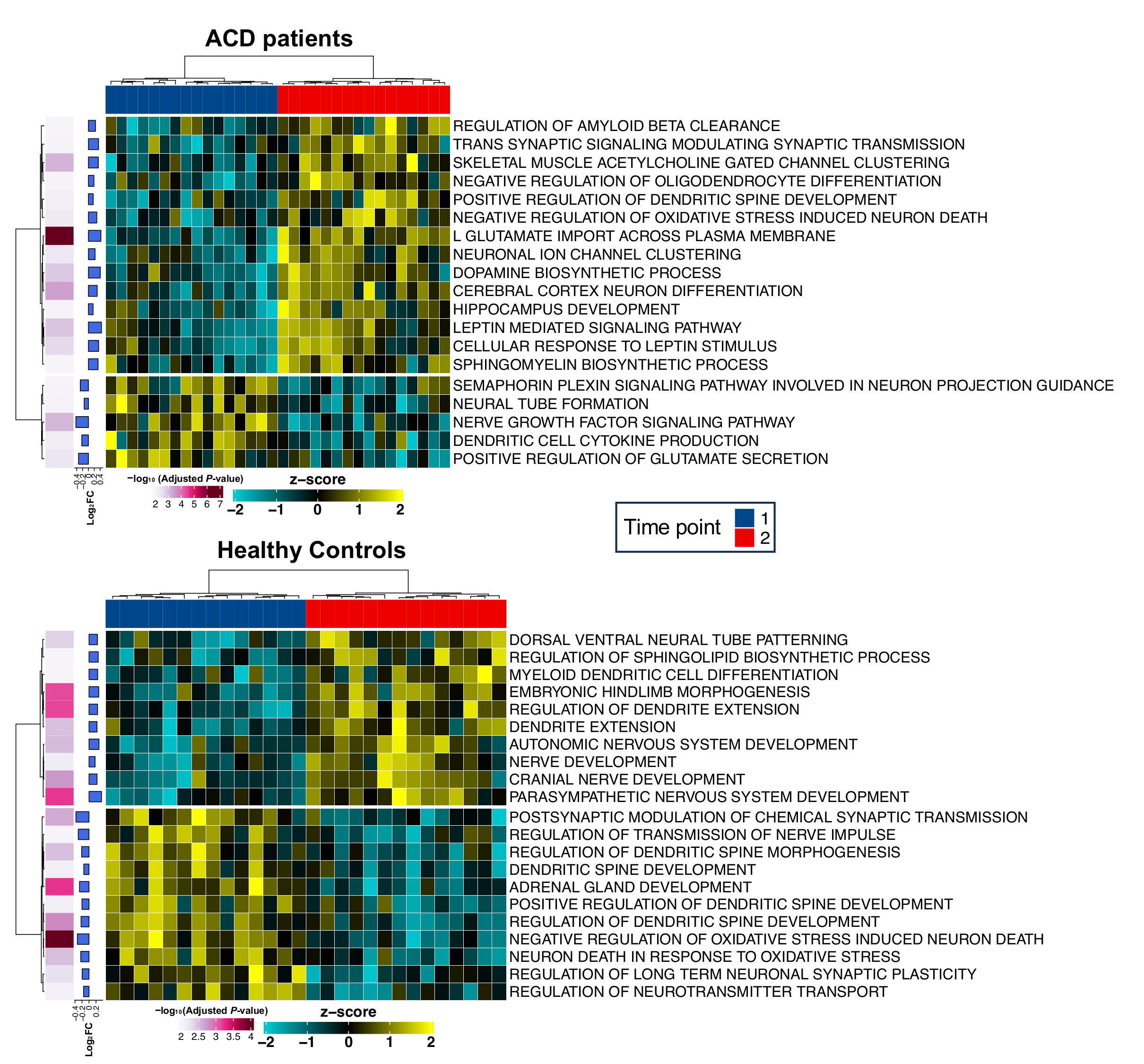

Supplement: Supplementary file 2 — Supplementary Figure S2. [file 41598_2023_48094_MOESM2_ESM.tif]

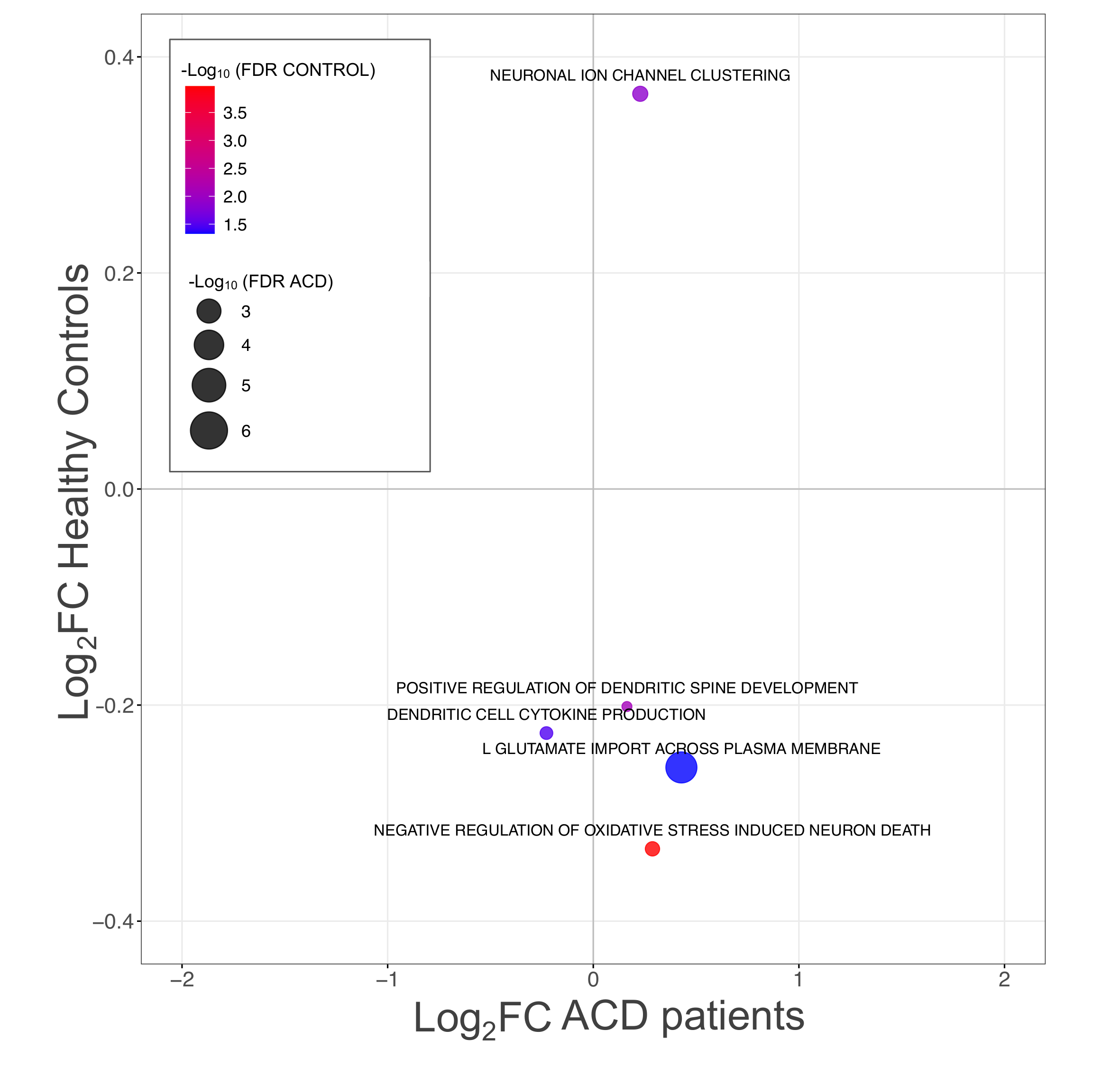

Supplement: Supplementary file 3 — Supplementary Figure S3. [file 41598_2023_48094_MOESM3_ESM.tif]

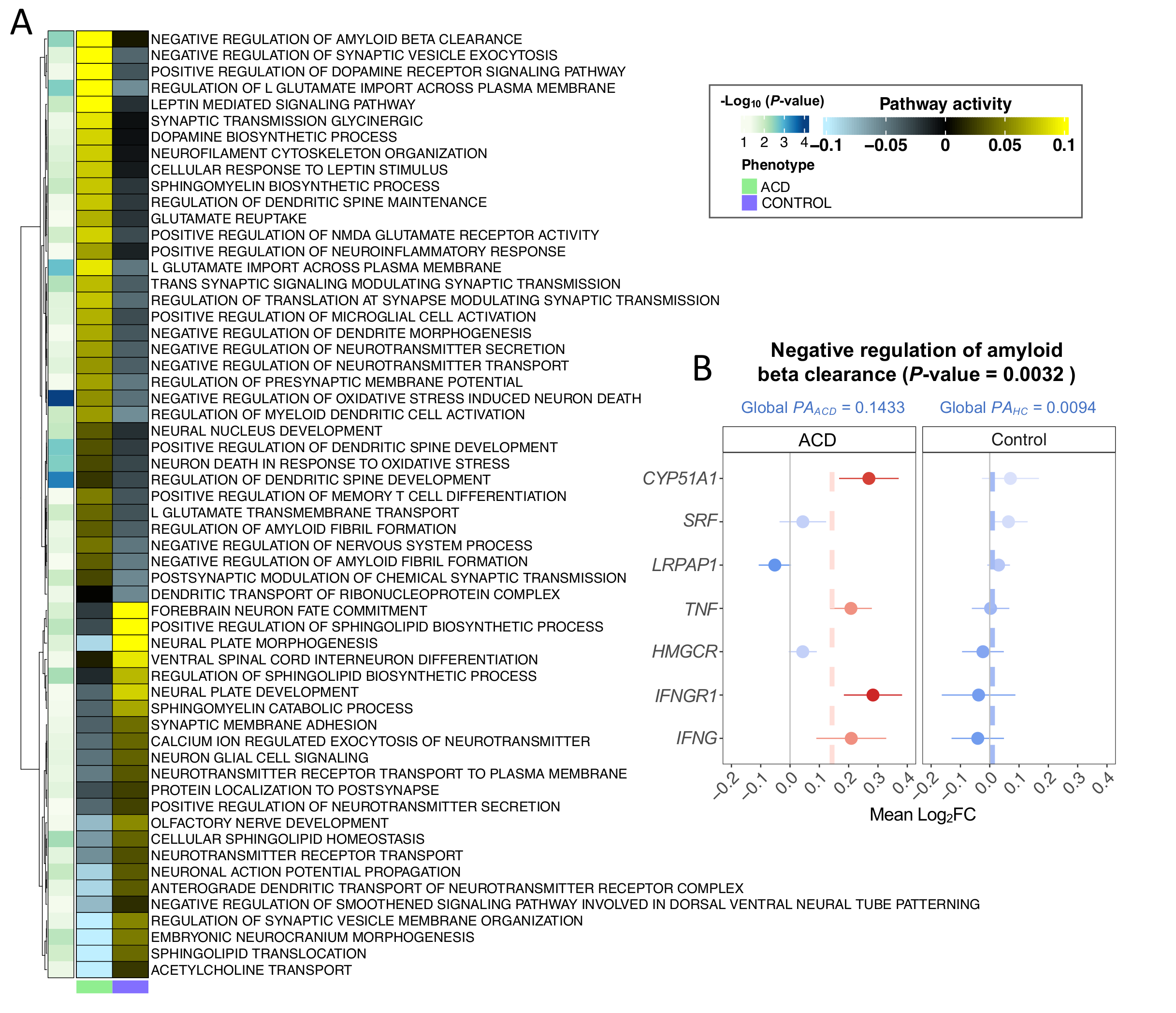

Supplement: Supplementary file 4 — Supplementary Figure S4. [file 41598_2023_48094_MOESM4_ESM.tif]

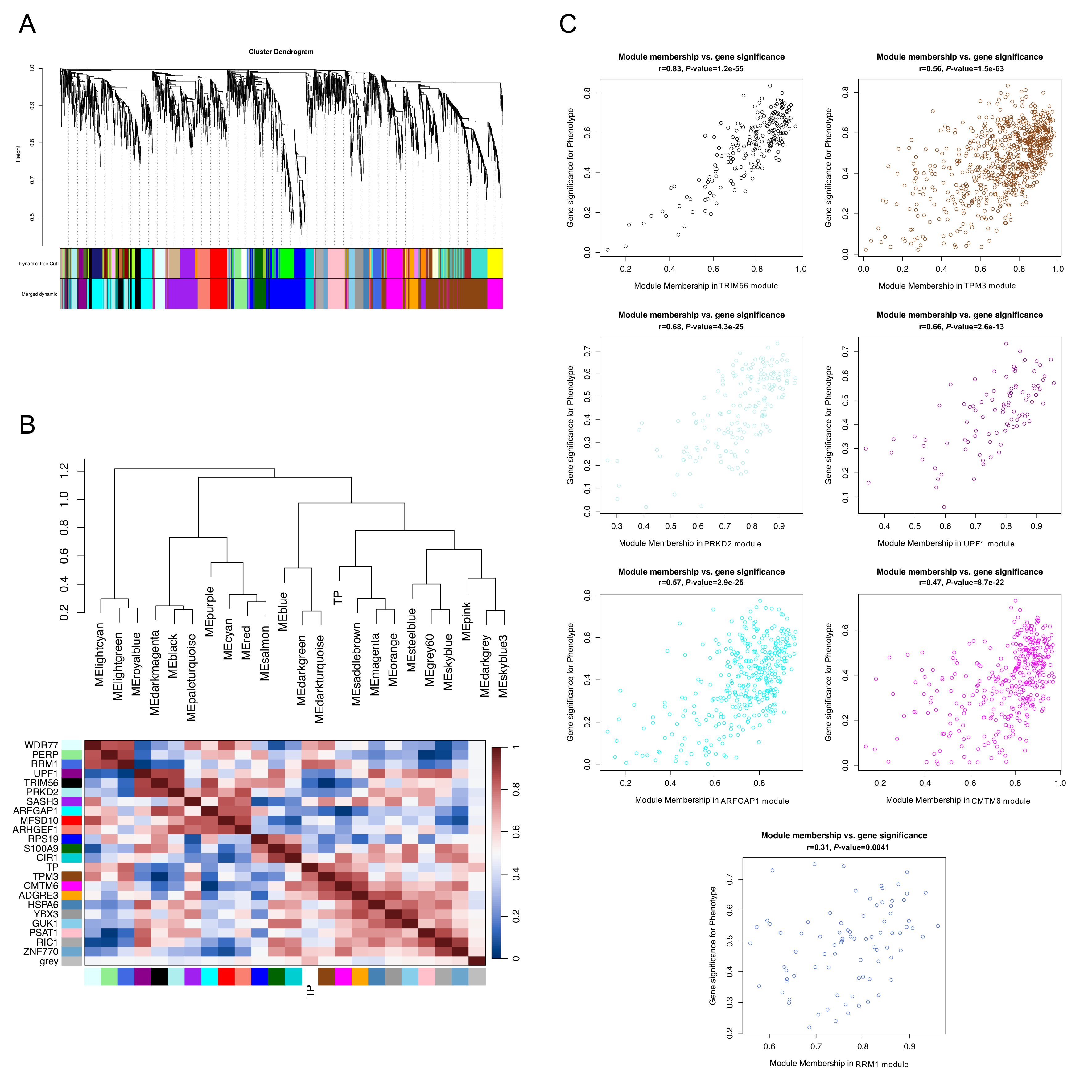

Supplement: Supplementary file 5 — Supplementary Figure S5. [file 41598_2023_48094_MOESM5_ESM.tif]

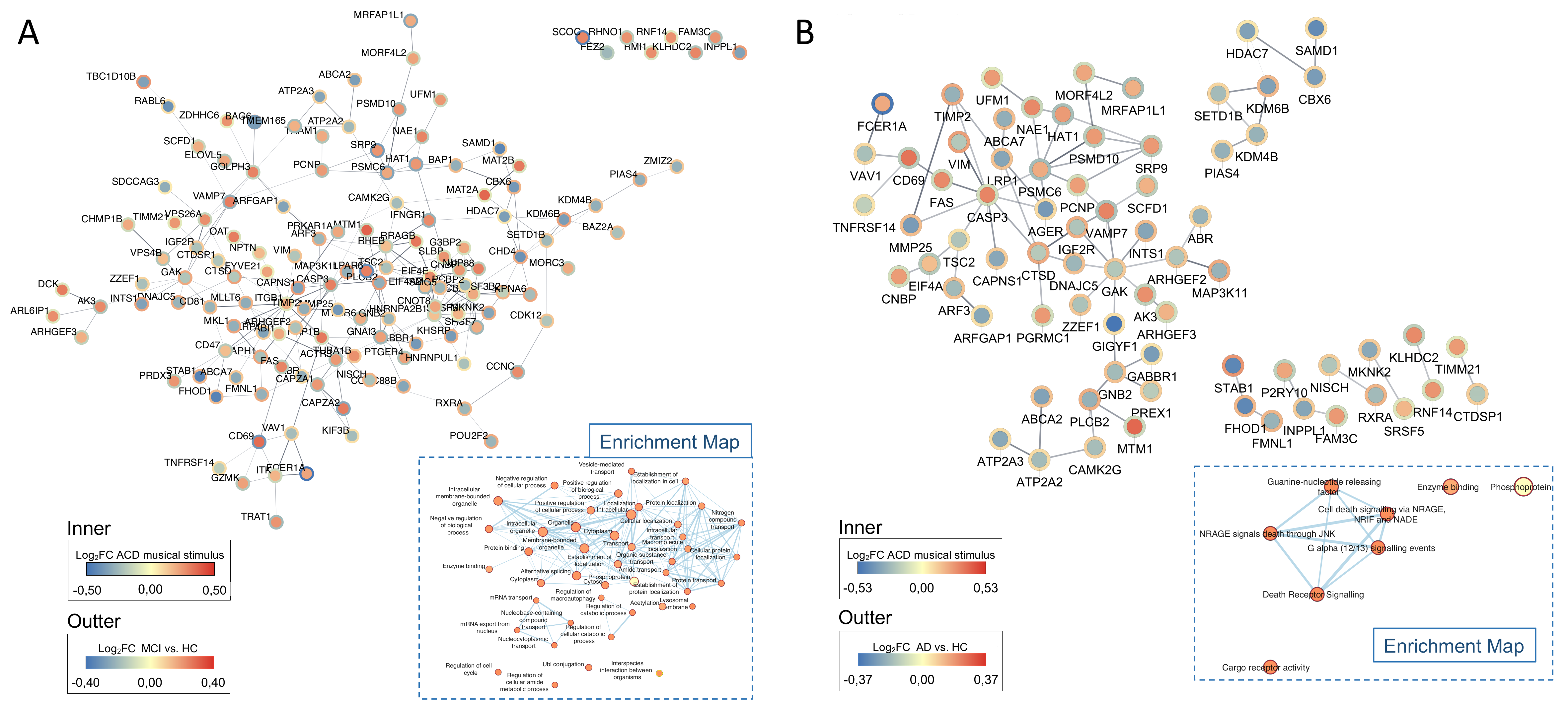

Supplement: Supplementary file 6 — Supplementary Figure S6. [file 41598_2023_48094_MOESM6_ESM.tif]

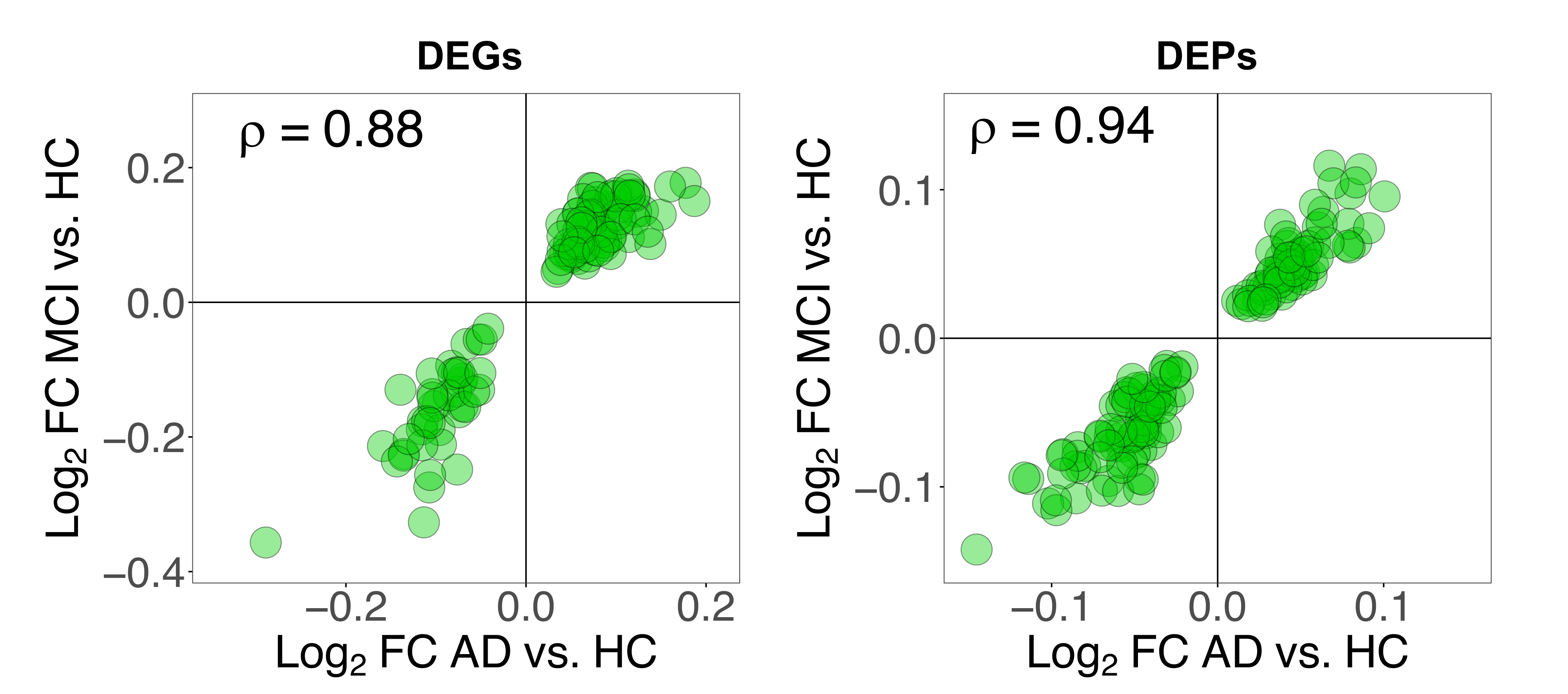

Supplement: Supplementary file 7 — Supplementary Figure S7. [file 41598_2023_48094_MOESM7_ESM.tif]
