# Supplementary material for: Music compensates for altered gene expression in age-related cognitive disorders
Source: Sci Rep. 2023 Dec 2;13:21259. doi: 10.1038/s41598-023-48094-5 (PMC10692168; doi:10.1038/s41598-023-48094-5)
Supplement: Supplementary file 8 — Supplementary Information 8. [file 41598_2023_48094_MOESM8_ESM.docx]

*Supplementary Note*

**Music compensates for altered gene expression in age-related cognitive disorders**

**Additional background**

Santiago Ramón y Cajal, who was awarded the Nobel Prize in Physiology and Medicine in 1906, and his colleagues affirmed that “*...the ability of a pianist … requires many years of mental and muscular gymnastics. To understand this important phenomenon, it is necessary to accept that … new pathways are created by the ramification and progressive growth of terminal dendritic and axonal processes*” [1]; Wan and Schlaug [2] have also quoted this statement. In the early days of research, the claim that music can induce changes in the brain’s anatomy was supported by limited and indirect evidence. However, current scientific evidence amply supports this proposition. For instance, Bermudez et al. [3] demonstrated that the thickness of the cortex, with peaks in the superior temporal and dorsolateral frontal regions, is greater in musicians compared to non-musician controls. In a review conducted by Wan and Schlaug [2], the existing evidence of music-making as a tool for promoting brain plasticity was analyzed, concluding that the human brain can be shaped by musical experiences. The review also pointed out the beneficial use of music as a non-pharmacological therapy in neurological and developmental disorders.

Furthermore, Fischer et al. [4] indicated that repeated exposure to personally meaningful music stimulates neural connectivity, leading to improvements in cognitive functioning in patients with early-stage cognitive decline. This finding suggests that music may have therapeutic applications in patients with dementia.

In addition, the emotional power of music has been a topic of interest since classical Greece, and especially during the Baroque period, where the doctrine of affections explored the relationship between music and emotions. Presently, there is scientific support for the neurological mechanisms underlying the ability of music to evoke powerful emotional responses [5]. Pereira et al. [6] used functional magnetic resonance imaging (fMRI) techniques to demonstrate that emotion-related limbic and paralimbic regions, as well as the reward circuitry, were more active when participants listened to familiar music compared to unfamiliar music. The study by Logeswaran and Bhattacharya [7] demonstrated that the emotions of music are cross-modal, such that they can spread from one sensory system to another. Additionally, using fMRI, Salimpoor et al. [8] demonstrated that neuronal activity in the mesolimbic striatum, particularly the nucleus accumbens, during the listening of a novel, unheard piece of music was the best predictor of the amount of money listeners were willing to spend on purchasing the piece. This study also highlighted the involvement of sensory cortical areas in reward processing.

In a recent systematic review on neuroscience and cognitive sciences, we [9] highlighted the existence of a global consensus on the beneficial effects of music on human health, particularly in relation to Alzheimer’s Disease (AD). According to this review, more than 93% of neuroscientific studies focused on music and neurodegenerative disease indicated a positive contribution of music to human health. The benefits of music were primarily associated with memory and cognition, as well as social behavior, mood, and emotion. This conclusion aligns with those of the World Health Organization (WHO). In a scoping review on health and well-being, the WHO recognized the growing evidence base for the role of the arts, including music, in improving health and well-being (https://www.who.int/europe/publications/i/item/9789289054553): *“the growing evidence base for the role of the arts in improving health and well-being by e.g. supporting the implementation of arts interventions where a substantial evidence base exists, such as the use of recorded music for patients prior to surgery, arts for patients with dementia and community arts programmes for mental health*”.

**Additional information on methods**

Participants, RNA isolation and RNA-Seq analysis

The experimental concert took place in the Auditorio de Galicia, which is renowned both nationally and internationally for hosting important classical music events. The participants in this study consisted on two cohorts: the first cohort comprised members of the ‘Asociación Galega de Axuda ós Enfermos con Demencias tipo Alzheimer’ (AGADEA) association, who suffer from age-related cognitive disorder (ACD). The second cohort served as the healthy control group and consisted of patient’s caregivers, and musicians. The study ensured that participants experienced a comfortable and welcoming environment during the classical music concert, minimizing exposure to stress factors that could potentially influence the experiment and bias the results. Several weeks prior to the Sensogenomics22-pilot concert, the project`s organizing team conducted informative sessions with donors at the mentioned auditorium, as well as at the Alzheimer’s Association offices and centers. AGADEA offers various local programs, resources, and services to patients, including home care, adult day care, and meal services. These sessions provided an opportunity to obtain written informed consent for sample collection. Additionally, a questionnaire was administered to collect data on the donors’ pathological conditions and self-reported music education. Neither ACD patients nor healthy controls reported hearing problems. Organizing these sessions well in advance of the experimental concert allowed for the prevention and minimization of potential stress factors on the day of the musical event, which could have negatively impacted the donors’ transcriptomes.

The musical repertoire lasted 50 minutes and included short pieces of classical music interpreted by different combinations of an initial septet (the SANARTE musical group, made of two violins, two violas, two cellos, and one horn) conformed by professional musicians (some were members of the Real Filharmonía de Galicia or RFG; www.rfgalicia.org). The repertoire consisted of adaptations of the “Spring” from the “Four Seasons” by Antonio Vivaldi, “A little night music, Allegro” by Wolfgang Amadeus Mozart, Pavane pour une Infante Defunte by Maurice Ravel, “La musica notturna delle strade di Madrid” by Luigi Boccherini, “Rondo in E-flat major, K.371” by Wolfgang Amadeus Mozart, the “Andante for Horn and Piano” by Richard Strauss, and “Por una cabeza” by Carlos Gardel. To prevent the audience (donors) from being influenced by the style and specific pieces of music being performed, the repertoire was not disclosed beforehand. The audience, including all donors, remained seated and listening to the musical stimuli without carrying out any physical activity.

A total of 100–200 μl of capillary blood was taken from all the donors using a capillary puncture procedure. The sample was first collected using a Minivette^®^ POCT K3 EDTA device and immediately transferred to microtubes containing an appropriate volume of PAXgene additive to stabilize the capillary blood and preserve RNA integrity. On average, the sample collection process lasted approximately 2–3 minutes for each donor, and all the samples were collected within two short time frames (less than 30 minutes) immediately before and after the concert.

RNA isolation and RNA-Seq analysis

Total RNA was isolated using the PAXgene blood miRNA extraction kit (Qiagen) adapting buffer volumes to the amount of blood collected (100–200μl). On-column DNase I treatment was carried out during the extraction process. RNA concentration step and an additional DNase treatment were undertaken using an RNA clean & concentrator kit (Zymo Research) prior to sequencing. The amount and integrity of the RNA obtained were checked using the TapeStation 4200 (Agilent). Input material was normalized to 10 ng, and strand specific library preparation was completed using the SMARTer Ultra low input RNA library (Poly-A). Sequencing was performed using a NovaSeq 6000 System at 100 paired end configuration to finally obtain 70M reads per sample. Quality control (QC) of the RNA-Seq data obtained was performed using the *FastQC* and *MultiQC* [10]. After the QC step, problematic 3' end and adapters were removed with the *Trimmomatic* package [11], and the reads mapped against the human reference genome (GCh38,v.104) using ultrafast universal RNA-Seq aligner *STAR* [12]. Secondary and low-quality alignments were removed using *SAMtools* [13]. To count the number of reads mapping to each gene (counts per gene), we used the software *HTSeq-counts* [14].

Pathway analysis

Biological pathways involved in musical stimulation were inferred directly from gene expression data following two different approaches. First, we used normalized data (corrected for paired sampling) as input in the Gene Set Variation Analysis (*GSVA*) R package [15] to detect biological processes that significantly change between baseline and TP2 in each of the groups separately (ACD patients and controls). We used the *gsva* method with a minimum gene-set size of 10 to estimate the gene-set enrichment scores per sample. Differential pathway expression analysis was carried out with *limma* [16]. This procedure was also employed to detect differentially expressed pathways (DEPs) in the AD case-control multi-cohort study. Second, two-way pathway analysis was performed using the Quantitative Set Analysis for Gene Expression (*QuSAGE*) R package [17] to find specific differential responses in biological processes to the musical stimuli between ACD patients and the control group. *QuSAGE* quantifies gene set activity using a complete Probability Density Function (PDF). As in the case of individual gene approach, we accounted for patient specific effects to calculate pathway activation by including sample information in the design formula. In both *GSVA* and *QuSAGE* analyses, we used the ontology gene-sets C5 (biological processes subset) as the reference database, contained in the file *gmt*, downloaded from the human Molecular Signatures DataBase (MSigDB) [18]. For some specific analysis, we used a sub-selection of neuro-biological processes by systematically and manually curating the pathway analysis results for items containing a neuro-biological term; this selection yielded a total of 483 biological processes (**Supplementary Note Table 1**).

Co-expression analysis

We investigated clusters of co-expressed genes potentially correlated to the musical stimuli in both ACD patients and the healthy donor group separately. Gene expression data normalized and corrected for patient-to-patient differences was used to build a signed weighted correlation network with the Weighted Gene Co-expression Network Analysis (*WGCNA*) R package [19]. Only genes that showed the most variant expression values between samples (the top 75% with the highest variance) were included in the analysis. A matrix of correlations between all pairs of selected genes was generated from the expression values, and further converted into an adjacency matrix with a power function. We chose a soft-thresholding power based on the criterion of scale-free topology after testing a set of candidate powers. A soft-thresholding power of 20 was selected as it resulted in the maximum model fitting index for both datasets (>0.85; **Supplementary Note Figure 1**). The Topological Overlap Matrix (TOM) and the corresponding dissimilarity (1–TOM) values were calculated. A minimum module size of 30, and 0.2 as the dendrogram cut height threshold for module merging were selected. The detected modules of co-expressed genes were labelled by colors and used to calculate module eigengenes (the PC1 of the module). The correlation between module eigengenes and the clinical trait was analyzed to identify modules of interest significantly associated with the musical stimuli (gene significance, or GS). Module Membership (MM) was calculated as a measure of intramodular connectivity. The hub genes within the significant modules are those with the highest connectivity; we named the modules by their most significant Hub genes.

We studied the biological significance of the correlated modules by performing an over-representation analysis and using the compare cluster function included in the *Clusterprofiler* R package [20] and the biological processes terms from the Gene Ontology (GO) database [21].

**Additional results**

Impact of music stimuli regarding previously reported music-related candidate genes

There is a set of candidate genes (*n* = 334) that have been previously reported to be related to music; their association is mainly inferred from genome association and transcriptomic studies [9]. We cross-compared this set of genes with those that appear as DE when contrasting the two time points. A total of 48 (out of 334; 14.4%; **Supplementary Note Table 2**) music-related genes appear as DEGs in TP2 *vs*. TP1 in ACD patients; among the most significant DEGs (those surpassing the multiple test adjustment) are (**Supplementary Note Figure 2A**): *PABPC1* (*P*-value = 3.5×10^-9^), *ADRB2* (*P*-value = 1.6×10^-4^), *GZMK* (*P*-value = 4.6×10^-7^), *CASP3* (*P*-value = 4.6×10^-5^), *APOBR* (*P*-value = 5.3×10^-6^), *JKAMP* (*P*-value = 1.1×10^-4^), *RNF213* (*P*-value = 6.9×10^-6^), *NPTN* (*P*-value = 9.1×10^-7^), *HDAC4* (*P*-value = 1.1×10^-4^), and *TFG* (*P*-value = 1.9×10^-5^). When examining controls, we detected 25 music-related genes (7.5%) that appear as DEGs in TP1 *vs*. TP2, with *GATA2* (see **Figure 2E** from main text) being the only one surpassing the multiple test adjustment.

DEGs and neuro-biological processes

To better investigate the nature of the DEGs emerging from the TP1 *vs*. TP2 comparison in ACD patients and controls, we examined GO biological terms related to neuro-biological processes and the genes that are involved in these terms. There are 2,192 protein coding genes among the DEGs in ACD patients; of these, 479 fall into neural-related processes, representing 22% of the whole protein coding DEGs (TP1 *vs*. TP2). Ten DEGs in ACD patients falling within these terms showed *P*-values < 10^-5^: *GNPAT* (*P*-value = 6.7×10^-6^), *UFM1* (*P*-value = 9.5×10^-6^), *GCH1* (*P*-value = 1.9×10^-6^) being up-regulated in TP2 with respect to TP1, and *ARHGEF2* (*P*-value = 2.7×10^-6^), *LAMB1* (*P*-value = 8.0×10^-6^), *DNAJC5* (*P*-value = 6.7×10^-6^), *AKT2* (*P*-value = 8.0×10^-6^), *JAK3* (*P*-value = 9.5×10^-6^), *FHOD1* (*P*-value = 8.0×10^-6^), and *SETD5* (*P*-value = 1.5×10^-6^) down-regulated; **Supplementary Note** **Figure 2B**. In healthy controls, there are 176 DEGs (TP1 *vs*. TP2) falling into neural-related processes, representing 19% of the protein coding DEGs (*n* = 921). Five out of the 176 DEGs in these neural categories showed a *P*-value < 10^-5^: two of them were significantly up-regulated (*GATA2* and *CCR5*), and three significantly down-regulated after musical stimuli (*PLCB1*, *ALCAM* and *PRKG1*); **Supplementary Note** **Figure 2B**.

Modules of co-expressed genes associated to musical stimuli in healthy control samples

In the control dataset, 15 modules of co-expressed genes were detected (**Supplementary Note Figure 3A; Supplementary Note Figure 3B**; **Supplementary Table S3** cited in the main text), 3 of them showing a statistically significant correlation with the musical stimuli (although not significant after multiple test correction). Globally, *ITPK1* [midnight blue] module (R = 0.42, *P*-value = 0.025) and *NFKB2* [greenyellow] module (R = 0.45, *P*-value = 0.016) were positively correlated (**Supplementary Note Figure 3C**), whereas the most significant module in controls, the *ATP5F1E* [pink] module (*P*-value = 0.0083), was negatively correlated with the musical stimuli (R = -0.49; **Supplementary Table S3** cited in the main text) and, as observed in the module expression patterns of individual samples, values tend to be lower in TP2 with respect to TP1; **Supplementary Note Figure 3D**. The genes most inter-connected within the *ATP5F1E* module also showed a high significant correlation with the musical stimuli (R = 0.38, *P*-value =1.8×10^-7^); **Supplementary Note Figure 3E**. Functional assessment of genes included in the *ATP5F1E* module pointed to processes mainly involved in cytoplasmatic translation (adjusted *P*-value = 3.63×10^-31^), oxidative phosphorylation (adjusted *P*-value = 1.41×10^-11^) and related processes (aerobic respiration and ATP synthesis in the mitochondria), and ribosome biogenesis (adjusted *P*-value = 1.05×10^-5^); **Supplementary Table S3** cited in the main text; **Supplementary Note Figure 4A**. Although of much less importance, the positively correlated *ITPK1* module could also be linked to activation of T-cell pathways. Genes from the most significant pathways belonging to the *ATP5F1E* module showed high MM values; **Supplementary Note Figure 4B.**

**Additional discussion**

Differential expressed genes in ACD and healthy controls

*GIGYF1* (GRB10-interacting gyf protein 1)

The GRB10-interacting gyf protein 1 (*GIGYF1*) gene is associated with autism spectrum disorder (ASD). Chen et al. [22] indicated that *GIGYF1* deficiency in mice led to a reduction of upper layer cortical neurons accompanied by decreased proliferation and increased differentiation of neural progenitor cells; these authors found that ASD individuals with *GIGYF1* likely gene disruptive (LGD) mutations were less likely to have cognitive impairments. According to these findings, it might be most relevant to investigate if a down-regulated *GIGYF1* (driven in the present study by the musical stimuli) could have some impact on cognitive benefits. Remarkably, *GIGYF1* (together with other genes) is associated with rapamycin sensitive genes (and it has been included in a patented invention for the prevention, treatment, and diagnosis of AD, namely, Therapeutic targets for AD - PCT/GB2O13/051843). In animal models, rapamycin has demonstrated to be the most effective pharmacological treatment to target the aging process to increase life and health span [23], and it also allows to delay/reverse age-related diseases (in mice, rats, and companion dogs), e.g., cancers, cardiac dysfunction, kidney disease, obesity, cognitive decline, periodontal disease, macular degeneration, muscle loss, stem cell function, and immune senescence [24-26]. Aggregation of amyloid beta (Aβ) peptides is a key feature of AD.

*ADAMTSL4*

It has been reported that the DEG (TP1 *vs*. TP2) *ADAMTS4* in ACD encodes for a disintegrin and metalloproteinase with thrombospondin motifs 4 enzyme. This metalloproteinase generates N-truncated Aβ4-x species and marks oligodendrocytes as a source of amyloidogenic peptides in AD [27]. *ADAMTS4* was included in the AD-specific network inferred from genetic association and interactome studies [28]. It was found highly expressed in the hippocampus of children with Autism Spectrum Disorder [29].

*GATA2* (GATA Binding Protein 2)

In healthy controls, the GATA Binding Protein 2 (*GATA2*) was found to be up-regulated in healthy controls when contrasting TP2 *vs*. TP1. This gene encodes a member of the GATA family of zinc-finger transcription factors that regulates GABAergic neurogenesis and migration [30] in adults. It has been discussed several times in the context of music and in the very few transcriptomic studies reported to date [31-35]. Recent studies have identified *GATA2* as regulator of key genes associated with neurodegenerative diseases, such as vascular dementia [36] and AD [37]. It also plays an essential role activating the expression of the neuroglobin gene, which encodes for a protein related to neuroprotection and involved in mitigating the consequences of stroke and AD [38].

Differential expressed pathways in ACD and healthy controls

*Metabolism of L-glutamate*

L-glutamate is the most abundant free amino acid in a healthy brain and the CNS, and it is a major excitatory neurotransmitter in mammals [39] with a main role in neuronal plasticity and synapsis, and also implicated in memory and learning processes. Over 40% of neuronal synapses are glutamatergic. The glutamatergic system is thought to play a fundamental role in long-term potentiation (LTP), a mechanism underlying learning and memory storage in the hippocampus and neocortex. Deficient levels of glutamate have been reported in brains of AD patients [40]. Loss of glutamate activity correlates to clinical dementia and MCI [41], playing a role in the pathogenesis of AD since early states [42]. This decrease in glutamate especially impacts on the posterior cingulate cortex, a region involved in cerebral glucose metabolism and beta-amyloid deposition in MCI [41]. The data suggest that the musical stimuli could contribute to managing L-glutamate in the brains of ACD patients, a target shared with traditional pharmacological therapies. Thus, among the most common current treatments aimed at reducing cognitive decline in AD are those targeting the cholinesterase and glutamatergic systems [43].

*Sphingolipidome and regulation of the Aβ peptides*

Another important difference between ACD and healthy controls is in the metabolism of sphingolipids. The complex universe of the sphingolipidome regulates a plethora of biological processes in cells. These molecules have kinases, phosphatase, and lipases as main targets to exert distinct cellular functions, and they have been found to be associated with neurodegenerative processes, as well as metabolic disorders, cancer, immune functions, and cardiovascular disorders [44]. An adequate regulation of sphingolipids metabolism in the brain is essential, such that subtle deviations of the sphingolipids balance might be sufficient to produce neurodegenerative conditions, such as AD or Parkinson [45]. Sphingolipids alteration can contribute to the neuropathological features of AD, including Aβ production, Tau formation and neurodegeneration. In this regard, sphingolipids metabolism correlated with Aβ levels in cerebrospinal fluid from AD patients [46] strengthening this effect of the sphingolipids balance disturbance in the Aβ clearance. Aβ in AD can produce membrane oxidative stress, resulting in accumulation of long-chain ceramides and cholesterol unleashing the clinical manifestations of the disease [47].

The accumulation of Aβ in the brain is an early toxic event in the pathogenesis of AD, and there is no efficient pharmacological treatment to stop it. The amyloid cascade hypothesis states that Aβ accumulation triggers tau hyperphosphorylation and aggregation; ultimately, these aggregates lead to inflammation, synaptic impairment, neuronal loss, and thus cognitive decline and behavioral abnormalities [48]. Even though our methodological approach cannot shed light on why music stimuli target on the regulation of Aβ (increasing the expression in ACD patients >15 times the expression of controls), it is a striking observation that music is able to impact on this key signature of neurodegeneration in AD patients. It is mandatory to investigate the relationship between this observation and the benefits of music reported by others in AD patients [9].

Co-expression networks

*Autophagic impairment*

Autophagic impairment has been related to neurodegenerative processes since it regulates intracytoplasmic levels of aggregate-prone proteins responsible for common neurodegenerative diseases, such as AD, Parkinson disease, Huntington disease, amyotrophic lateral sclerosis, or dementia [49-52]. Aggregates of Tau and Aβ as well as accumulation of autophagosomes in neurons of AD patients are clear signals of autophagic system disruption. Autophagy is a key regulator of Aβ generation and clearance, as Aβ is released from neurons through an autophagy-dependent mechanism [53]. Moreover, accumulation of autophagosomes (autophagosomes and autolysosomes [autophagic vacuoles]) has been observed in neurons from AD patients in different stages [54, 55]. However, there are discrepancies in the literature as to whether an up-regulation of autophagic activity is responsible for the accumulation of these autophagosomes [56] or, conversely, whether this accumulation might be due to a decrease in autophagic-mediated protein release or autophagosome clearance activity [55, 57, 58]. Recent studies claim that autophagy can be differentially affected at different stages of AD [53, 59]. Currently, different autophagy modulators are being investigated as treatments for AD [60, 61].

Enrichment map

The Receptor for advanced glycation end products (RAGE) is a multiligand transmembrane receptor of the immunoglobulin superfamily. It is highly expressed in neurons of patients suffering neurodegenerative diseases and is also significantly up-regulated in AD-related optic neuropathy [62]. RAGE transports the Aβ toxins through the blood-brain barrier; however, in AD, RAGE mediates Aβ toxicity, disrupting the blood-brain barrier and promoting AD occurrence and progression [63, 64]. The up-regulation of RAGE accelerates Aβ uptake and transport, facilitating blood-brain barrier crossing *via* endocytosis, activating inflammatory pathways, oxidative stress, neuroinflammation, and synaptic and neuronal destruction [65, 66]. Moreover, accumulation of Aβ oligomers can also induce RAGE-mediated autophagy, interfering with the proper functioning of tight junction proteins in the blood-brain barrier and, thus, contributing to the worsening of AD [67, 68]. Due to its role in MCI/AD pathogenesis, RAGE is being investigated as a therapeutic target in AD [69]; reviewed in [70].

Limitations

There are various limitations to the present study. First, its design does not allow to completely isolate the music stimuli from other possible stimuli and confounding factors. This fact, however, characterizes most (if not all) studies targeting complex multifactorial traits e.g. [71-74]. It is the population-based nature of these discovery-based studies that minimizes the possible impact of individual features in transcriptomes (which could deviate from the ‘average’ due to possible confounded factors). Moreover, to minimize possible confounding effects, the experimental concert Sensogenomics22-pilot was conceived to reduce potential noise factors as much as possible. For instance, the procedures for sample collection were minimally invasive (capillary puncture), with the aim of reducing possible stress factors, e.g., avoiding waiting times for patients that could develop anxiety or stress, accompaniment of patients by their caregivers, etc. In this regard, it has been reported that transcriptomes analyzed from fingerstick (capillary) blood samples (as done here) represent a very good proxy for transcriptomes analyzed from venipuncture (venous) blood [75, 76], although this issue might be controversial, at least in the context of vaccination [77]. In any case, it seems clear from our results that capillary blood samples capture transcriptomics changes occurring because of the simple impact of a sensorial stimulus. These changes are most likely echoing those occurring in the brain, as this is the natural anatomical entry point for the musical stimuli. In addition, the musical event took place in a natural place for music, as it would with any other non-experimental musical event. The ecological validity of the experiment adds extra value to the study; to the best of our knowledge, there is no comparable precedent in the scientific literature. Among other limitations, it is noteworthy to mention that, although we have investigated many more samples than previous experiments on the effect of music on transcriptomes, it is mandatory to validate them using much larger cohorts. Some of the analyses carried out in the present study involved comparing data obtained from different technologies to measure gene expression, namely, RNAseq and microarrays. However, comparing fold-changes from the same or different platforms should not be considered a significant limitation. Correlating fold-changes is a common and well-accepted practice when comparing results across various platforms, technologies, studies, or even various -omics datasets (as exemplified in Fig 5c of [78], Fig 6b in [79], or Fig 2b/e in [80]). This approach enables the analysis of the direction and magnitude of differential expression across different platforms or studies. Finally, we have detected more changes in the transcriptomes of ACD patients than in the transcriptomes of controls; given that the sample size of both cohorts was comparable, it is highly probable that the musical stimuli might have a higher impact on the transcriptomes of cases than in those from controls. In our study, there is an age difference between the ADC and the control group. While this difference may affect only the comparisons made between these two groups, it does not impact the analyses comparing different time points or those aimed at contrasting the effect of music on ADC against genes altered in AD/MCI patients. The latter comparisons, in fact, constitute the main findings of the present study.

A powerful study would be needed to validate the results of the present study and increase the probability to detect more changes occurring in the transcriptomes of healthy controls.

**References**

1. Ramon y Cajal S, Pasik P, Pasik F: **Texture of the nervous system of man and the vertebrates**: Springer-Verlag; New York; Original work published in 1904; 1999.

2. Wan CY, Schlaug G: **Music making as a tool for promoting brain plasticity across the life span**. *Neuroscientist* 2010, **16**(5):566-577.

3. Bermudez P, Lerch JP, Evans AC, Zatorre RJ: **Neuroanatomical correlates of musicianship as revealed by cortical thickness and voxel-based morphometry**. *Cereb Cortex* 2009, **19**(7):1583-1596.

4. Fischer CE, Churchill N, Leggieri M, Vuong V, Tau M, Fornazzari LR, Thaut MH, Schweizer TA: **Long-Known Music Exposure Effects on Brain Imaging and Cognition in Early-Stage Cognitive Decline: A Pilot Study**. *Journal of Alzheimer's disease : JAD* 2021, **84**(2):819-833.

5. Jäncke L: **Music, memory and emotion**. *J Biol* 2008, **7**(6):21.

6. Pereira CS, Teixeira J, Figueiredo P, Xavier J, Castro SL, Brattico E: **Music and emotions in the brain: familiarity matters**. *PLoS One* 2011, **6**(11):e27241.

7. Logeswaran N, Bhattacharya J: **Crossmodal transfer of emotion by music**. *Neurosci Lett* 2009, **455**(2):129-133.

8. Salimpoor VN, van den Bosch I, Kovacevic N, McIntosh AR, Dagher A, Zatorre RJ: **Interactions between the nucleus accumbens and auditory cortices predict music reward value**. *Science* 2013, **340**(6129):216-219.

9. Navarro L, Gomez-Carballa A, Pischedda S, Montoto-Louzao J, Viz-Lasheras S, Camino-Mera A, Hinault T, Martinon-Torres F, Salas A: **Sensogenomics of music and Alzheimer's disease: An interdisciplinary view from neuroscience, transcriptomics, and epigenomics**. *Front Aging Neurosci* 2023, **15**:1063536.

10. Ewels P, Magnusson M, Lundin S, Kaller M: **MultiQC: summarize analysis results for multiple tools and samples in a single report**. *Bioinformatics* 2016, **32**(19):3047-3048.

11. Bolger AM, Lohse M, Usadel B: **Trimmomatic: a flexible trimmer for Illumina sequence data**. *Bioinformatics* 2014, **30**(15):2114-2120.

12. Dobin A, Davis CA, Schlesinger F, Drenkow J, Zaleski C, Jha S, Batut P, Chaisson M, Gingeras TR: **STAR: ultrafast universal RNA-seq aligner**. *Bioinformatics* 2013, **29**(1):15-21.

13. Danecek P, Auton A, Abecasis G, Albers CA, Banks E, DePristo MA, Handsaker RE, Lunter G, Marth GT, Sherry ST *et al*: **The variant call format and VCFtools**. *Bioinformatics* 2011, **27**(15):2156-2158.

14. Anders S, Pyl PT, Huber W: **HTSeq--a Python framework to work with high-throughput sequencing data**. *Bioinformatics* 2015, **31**(2):166-169.

15. Hänzelmann S, Castelo R, Guinney J: **GSVA: gene set variation analysis for microarray and RNA-seq data**. *BMC Bioinformatics* 2013, **14**:7.

16. Ritchie ME, Phipson B, Wu D, Hu Y, Law CW, Shi W, Smyth GK: ***limma* powers differential expression analyses for RNA-sequencing and microarray studies**. *Nucleic Acids Res* 2015, **43**(7):e47.

17. Meng H, Yaari G, Bolen CR, Avey S, Kleinstein SH: **Gene set meta-analysis with Quantitative Set Analysis for Gene Expression (QuSAGE)**. *PLoS computational biology* 2019, **15**(4):e1006899.

18. Liberzon A, Birger C, Thorvaldsdottir H, Ghandi M, Mesirov JP, Tamayo P: **The Molecular Signatures Database (MSigDB) hallmark gene set collection**. *Cell Syst* 2015, **1**(6):417-425.

19. Langfelder P, Horvath S: **WGCNA: an R package for weighted correlation network analysis**. *BMC Bioinformatics* 2008, **9**:559.

20. Wu T, Hu E, Xu S, Chen M, Guo P, Dai Z, Feng T, Zhou L, Tang W, Zhan L *et al*: **clusterProfiler 4.0: A universal enrichment tool for interpreting omics data**. *Innovation (N Y)* 2021, **2**(3):100141.

21. Mi H, Huang X, Muruganujan A, Tang H, Mills C, Kang D, Thomas PD: **PANTHER version 11: expanded annotation data from Gene Ontology and Reactome pathways, and data analysis tool enhancements**. *Nucleic Acids Res* 2017, **45**(D1):D183-D189.

22. Chen G, Yu B, Tan S, Tan J, Jia X, Zhang Q, Zhang X, Jiang Q, Hua Y, Han Y *et al*: **GIGYF1 disruption associates with autism and impaired IGF-1R signaling**. *The Journal of clinical investigation* 2022, **132**(19).

23. Johnson SC, Sangesland M, Kaeberlein M, Rabinovitch PS: **Modulating mTOR in aging and health**. *Interdiscip Top Gerontol* 2015, **40**:107-127.

24. Johnson SC, Martin GM, Rabinovitch PS, Kaeberlein M: **Preserving youth: does rapamycin deliver?** *Sci Transl Med* 2013, **5**(211):211fs240.

25. Kennedy BK, Lamming DW: **The Mechanistic Target of Rapamycin: The Grand ConducTOR of Metabolism and Aging**. *Cell Metab* 2016, **23**(6):990-1003.

26. Kaeberlein M, Galvan V: **Rapamycin and Alzheimer's disease: Time for a clinical trial?** *Sci Transl Med* 2019, **11**(476).

27. Walter S, Jumpertz T, Huttenrauch M, Ogorek I, Gerber H, Storck SE, Zampar S, Dimitrov M, Lehmann S, Lepka K *et al*: **The metalloprotease ADAMTS4 generates N-truncated Abeta4-x species and marks oligodendrocytes as a source of amyloidogenic peptides in Alzheimer's disease**. *Acta Neuropathol* 2019, **137**(2):239-257.

28. Hu YS, Xin J, Hu Y, Zhang L, Wang J: **Analyzing the genes related to Alzheimer's disease via a network and pathway-based approach**. *Alzheimers Res Ther* 2017, **9**(1):29.

29. Rexrode L, Hartley J, Showmaker KC, Vandewege MW, Martin BE, Blair E, Bollavarapu R, Antonyraj RB, Hilton K, Gardiner A *et al*: **Molecular Profiling of the Hippocampus of Children with Autism Spectrum Disorder**. *medRxiv* 2023, **doi.org/10.1101/2022.10.13.22281011**.

30. Achim K, Salminen M, Partanen J: **Mechanisms regulating GABAergic neuron development**. *Cellular and molecular life sciences : CMLS* 2014, **71**(8):1395-1415.

31. Nair PS, Raijas P, Ahvenainen M, Philips AK, Ukkola-Vuoti L, Jarvela I: **Music-listening regulates human microRNA expression**. *Epigenetics* 2020:1-13.

32. Järvelä I: **Genomics studies on musical aptitude, music perception, and practice**. *Ann N Y Acad Sci* 2018.

33. Kanduri C, Raijas P, Ahvenainen M, Philips AK, Ukkola-Vuoti L, Lahdesmaki H, Jarvela I: **The effect of listening to music on human transcriptome**. *PeerJ* 2015, **3**:e830.

34. Szyfter K, Witt MP: **How far musicality and perfect pitch are derived from genetic factors?** *Journal of applied genetics* 2020, **61**(3):407-414.

35. Oikkonen J, Huang Y, Onkamo P, Ukkola-Vuoti L, Raijas P, Karma K, Vieland VJ, Jarvela I: **A genome-wide linkage and association study of musical aptitude identifies loci containing genes related to inner ear development and neurocognitive functions**. *Mol Psychiatry* 2015, **20**(2):275-282.

36. Shu J, Wei W, Zhang L: **Identification of Molecular Signatures and Candidate Drugs in Vascular Dementia by Bioinformatics Analyses**. *Front Mol Neurosci* 2022, **15**:751044.

37. Rahman MR, Islam T, Zaman T, Shahjaman M, Karim MR, Huq F, Quinn JMW, Holsinger RMD, Gov E, Moni MA: **Identification of molecular signatures and pathways to identify novel therapeutic targets in Alzheimer's disease: Insights from a systems biomedicine perspective**. *Genomics* 2020, **112**(2):1290-1299.

38. Tam KT, Chan PK, Zhang W, Law PP, Tian Z, Fung Chan GC, Philipsen S, Festenstein R, Tan-Un KC: **Identification of a novel distal regulatory element of the human Neuroglobin gene by the chromosome conformation capture approach**. *Nucleic Acids Res* 2017, **45**(1):115-126.

39. Zhou Y, Danbolt NC: **Glutamate as a neurotransmitter in the healthy brain**. *J Neural Transm (Vienna)* 2014, **121**(8):799-817.

40. Fayed N, Modrego PJ, Rojas-Salinas G, Aguilar K: **Brain glutamate levels are decreased in Alzheimer's disease: a magnetic resonance spectroscopy study**. *Am J Alzheimers Dis Other Demen* 2011, **26**(6):450-456.

41. Oeltzschner G, Wijtenburg SA, Mikkelsen M, Edden RAE, Barker PB, Joo JH, Leoutsakos JS, Rowland LM, Workman CI, Smith GS: **Neurometabolites and associations with cognitive deficits in mild cognitive impairment: a magnetic resonance spectroscopy study at 7 Tesla**. *Neurobiol Aging* 2019, **73**:211-218.

42. Esposito Z, Belli L, Toniolo S, Sancesario G, Bianconi C, Martorana A: **Amyloid beta, glutamate, excitotoxicity in Alzheimer's disease: are we on the right track?** *CNS Neurosci Ther* 2013, **19**(8):549-555.

43. Conway ME: **Alzheimer's disease: targeting the glutamatergic system**. *Biogerontology* 2020, **21**(3):257-274.

44. Hannun YA, Obeid LM: **Sphingolipids and their metabolism in physiology and disease**. *Nat Rev Mol Cell Biol* 2018, **19**(3):175-191.

45. Mielke MM, Lyketsos CG: **Alterations of the sphingolipid pathway in Alzheimer's disease: new biomarkers and treatment targets?** *Neuromolecular Med* 2010, **12**(4):331-340.

46. Fonteh AN, Ormseth C, Chiang J, Cipolla M, Arakaki X, Harrington MG: **Sphingolipid metabolism correlates with cerebrospinal fluid Beta amyloid levels in Alzheimer's disease**. *PLoS One* 2015, **10**(5):e0125597.

47. Cutler RG, Kelly J, Storie K, Pedersen WA, Tammara A, Hatanpaa K, Troncoso JC, Mattson MP: **Involvement of oxidative stress-induced abnormalities in ceramide and cholesterol metabolism in brain aging and Alzheimer's disease**. *Proc Natl Acad Sci U S A* 2004, **101**(7):2070-2075.

48. Roda AR, Serra-Mir G, Montoliu-Gaya L, Tiessler L, Villegas S: **Amyloid-beta peptide and tau protein crosstalk in Alzheimer's disease**. *Neural Regen Res* 2022, **17**(8):1666-1674.

49. Ravikumar B, Duden R, Rubinsztein DC: **Aggregate-prone proteins with polyglutamine and polyalanine expansions are degraded by autophagy**. *Hum Mol Genet* 2002, **11**(9):1107-1117.

50. Webb JL, Ravikumar B, Atkins J, Skepper JN, Rubinsztein DC: **Alpha-Synuclein is degraded by both autophagy and the proteasome**. *The Journal of biological chemistry* 2003, **278**(27):25009-25013.

51. Barmada SJ, Serio A, Arjun A, Bilican B, Daub A, Ando DM, Tsvetkov A, Pleiss M, Li X, Peisach D *et al*: **Autophagy induction enhances TDP43 turnover and survival in neuronal ALS models**. *Nat Chem Biol* 2014, **10**(8):677-685.

52. Berger Z, Ravikumar B, Menzies FM, Oroz LG, Underwood BR, Pangalos MN, Schmitt I, Wullner U, Evert BO, O'Kane CJ *et al*: **Rapamycin alleviates toxicity of different aggregate-prone proteins**. *Hum Mol Genet* 2006, **15**(3):433-442.

53. Nilsson P, Saido TC: **Dual roles for autophagy: degradation and secretion of Alzheimer's disease Abeta peptide**. *Bioessays* 2014, **36**(6):570-578.

54. Nixon RA, Wegiel J, Kumar A, Yu WH, Peterhoff C, Cataldo A, Cuervo AM: **Extensive involvement of autophagy in Alzheimer disease: an immuno-electron microscopy study**. *J Neuropathol Exp Neurol* 2005, **64**(2):113-122.

55. Boland B, Kumar A, Lee S, Platt FM, Wegiel J, Yu WH, Nixon RA: **Autophagy induction and autophagosome clearance in neurons: relationship to autophagic pathology in Alzheimer's disease**. *J Neurosci* 2008, **28**(27):6926-6937.

56. Lipinski MM, Zheng B, Lu T, Yan Z, Py BF, Ng A, Xavier RJ, Li C, Yankner BA, Scherzer CR *et al*: **Genome-wide analysis reveals mechanisms modulating autophagy in normal brain aging and in Alzheimer's disease**. *Proc Natl Acad Sci U S A* 2010, **107**(32):14164-14169.

57. Lee S, Sato Y, Nixon RA: **Lysosomal proteolysis inhibition selectively disrupts axonal transport of degradative organelles and causes an Alzheimer's-like axonal dystrophy**. *J Neurosci* 2011, **31**(21):7817-7830.

58. Pickford F, Masliah E, Britschgi M, Lucin K, Narasimhan R, Jaeger PA, Small S, Spencer B, Rockenstein E, Levine B *et al*: **The autophagy-related protein beclin 1 shows reduced expression in early Alzheimer disease and regulates amyloid beta accumulation in mice**. *The Journal of clinical investigation* 2008, **118**(6):2190-2199.

59. Frake RA, Ricketts T, Menzies FM, Rubinsztein DC: **Autophagy and neurodegeneration**. *The Journal of clinical investigation* 2015, **125**(1):65-74.

60. Liu J, Li L: **Targeting Autophagy for the Treatment of Alzheimer's Disease: Challenges and Opportunities**. *Front Mol Neurosci* 2019, **12**:203.

61. Uddin MS, Stachowiak A, Mamun AA, Tzvetkov NT, Takeda S, Atanasov AG, Bergantin LB, Abdel-Daim MM, Stankiewicz AM: **Autophagy and Alzheimer's Disease: From Molecular Mechanisms to Therapeutic Implications**. *Front Aging Neurosci* 2018, **10**:04.

62. Wang MY, Ross-Cisneros FN, Aggarwal D, Liang CY, Sadun AA: **Receptor for advanced glycation end products is upregulated in optic neuropathy of Alzheimer's disease**. *Acta Neuropathol* 2009, **118**(3):381-389.

63. Yan SD, Chen X, Fu J, Chen M, Zhu H, Roher A, Slattery T, Zhao L, Nagashima M, Morser J *et al*: **RAGE and amyloid-beta peptide neurotoxicity in Alzheimer's disease**. *Nature* 1996, **382**(6593):685-691.

64. Cai Z, Liu N, Wang C, Qin B, Zhou Y, Xiao M, Chang L, Yan LJ, Zhao B: **Role of RAGE in Alzheimer's Disease**. *Cell Mol Neurobiol* 2016, **36**(4):483-495.

65. Fang F, Yu Q, Arancio O, Chen D, Gore SS, Yan SS, Yan SF: **RAGE mediates Abeta accumulation in a mouse model of Alzheimer's disease via modulation of beta- and gamma-secretase activity**. *Hum Mol Genet* 2018, **27**(6):1002-1014.

66. Deane R, Zlokovic BV: **Role of the blood-brain barrier in the pathogenesis of Alzheimer's disease**. *Curr Alzheimer Res* 2007, **4**(2):191-197.

67. Chan Y, Chen W, Wan W, Chen Y, Li Y, Zhang C: **Abeta(1-42) oligomer induces alteration of tight junction scaffold proteins via RAGE-mediated autophagy in bEnd.3 cells**. *Exp Cell Res* 2018, **369**(2):266-274.

68. Son SM, Jung ES, Shin HJ, Byun J, Mook-Jung I: **Abeta-induced formation of autophagosomes is mediated by RAGE-CaMKKbeta-AMPK signaling**. *Neurobiol Aging* 2012, **33**(5):1006 e1011-1023.

69. Kinscherf NA, Pehar M: **Role and Therapeutic Potential of RAGE Signaling in Neurodegeneration**. *Curr Drug Targets* 2022, **23**(12):1191-1209.

70. Deane RJ: **Is RAGE still a therapeutic target for Alzheimer's disease?** *Future Med Chem* 2012, **4**(7):915-925.

71. Butler-Laporte G, Povysil G, Kosmicki JA, Cirulli ET, Drivas T, Furini S, Saad C, Schmidt A, Olszewski P, Korotko U *et al*: **Exome-wide association study to identify rare variants influencing COVID-19 outcomes: Results from the Host Genetics Initiative**. *PLoS Genet* 2022, **18**(11):e1010367.

72. Jones E, Hummerich H, Vire E, Uphill J, Dimitriadis A, Speedy H, Campbell T, Norsworthy P, Quinn L, Whitfield J *et al*: **Identification of novel risk loci and causal insights for sporadic Creutzfeldt-Jakob disease: a genome-wide association study**. *Lancet neurology* 2020, **19**(10):840-848.

73. Kumar V, Pouw RB, Autio MI, Sagmeister MG, Phua ZY, Borghini L, Wright VJ, Hoggart C, Pan B, Tan AKY *et al*: **Variation in CFHR3 determines susceptibility to meningococcal disease by controlling factor H concentrations**. *Am J Hum Genet* 2022, **109**(9):1680-1691.

74. Aung T, Ozaki M, Lee MC, Schlotzer-Schrehardt U, Thorleifsson G, Mizoguchi T, Igo RP, Jr., Haripriya A, Williams SE, Astakhov YS *et al*: **Genetic association study of exfoliation syndrome identifies a protective rare variant at *LOXL1* and five new susceptibility loci**. *Nat Genet* 2017, **49**(7):993-1004.

75. Toma R, Duval N, Pelle B, Parks MM, Gopu V, Torres PJ, Camacho FR, Shen N, Krishnan S, Hatch A *et al*: **A clinically validated human capillary blood transcriptome test for global systems biology studies**. *Biotechniques* 2020, **69**(4):289-301.

76. Robison EH, Mondala TS, Williams AR, Head SR, Salomon DR, Kurian SM: **Whole genome transcript profiling from fingerstick blood samples: a comparison and feasibility study**. *BMC Genomics* 2009, **10**:617.

77. Stein DF, O'Connor D, Blohmke CJ, Sadarangani M, Pollard AJ: **Gene expression profiles are different in venous and capillary blood: Implications for vaccine studies**. *Vaccine* 2016, **34**(44):5306-5313.

78. Consortium SM-I: **A comprehensive assessment of RNA-seq accuracy, reproducibility and information content by the Sequencing Quality Control Consortium**. *Nature biotechnology* 2014, **32**(9):903-914.

79. Coleman SR, Smith ML, Spicer V, Lao Y, Mookherjee N, Hancock REW: **Overexpression of the Small RNA PA0805.1 in Pseudomonas aeruginosa Modulates the Expression of a Large Set of Genes and Proteins, Resulting in Altered Motility, Cytotoxicity, and Tobramycin Resistance**. *mSystems* 2020, **5**(3).

80. Kogenaru S, Qing Y, Guo Y, Wang N: **RNA-seq and microarray complement each other in transcriptome profiling**. *BMC Genomics* 2012, **13**:629.

**Supplementary Note Figure 1.** Soft-thresholding power estimation in ACD and healthy controls cohorts. Plots represent correlation between soft-thresholding powers and both the scale-free fit index and the mean connectivity.

**Supplementary Note Figure 2**. (A) Music-related DEGs in ACD patients when comparing TP1 *vs*. TP2 (adjusted *P*-value <0.05), and (B) DEGs involved in neuronal-related processes (*P*-values < 10^-5^) in ACD patients and controls when comparing when comparing TP1 *vs*. TP2.

**Supplementary Note Figure 3.** Co-expression network analysis in healthy control cohort. (A) Clustering dendrogram of genes and co-expression modules detected represented by different colors. (B) Correlation values heatmap obtained from WGCNA module analysis of co-expressed genes in healthy controls. Upper value shows to individual correlation value of the module with musical stimuli. *P*-values of these correlations are represented in brackets (lower values). (C) Hierarchical clustering eigengene dendrogram and heatmap for healthy controls dataset showing relationships among the modules and TP (musical stimuli; TP2 *vs.* TP1). Gene names on the left of the heatmap are the hub genes of each module. (D) Raincloud plots of differences in samples eigengene values between TP1 and TP2 from modules showing the largest statistical significance in healthy controls. (E) Plot showing comparison between MM (module membership) and musical stimuli correlation of genes from the most significant module detected in the healthy control cohort.


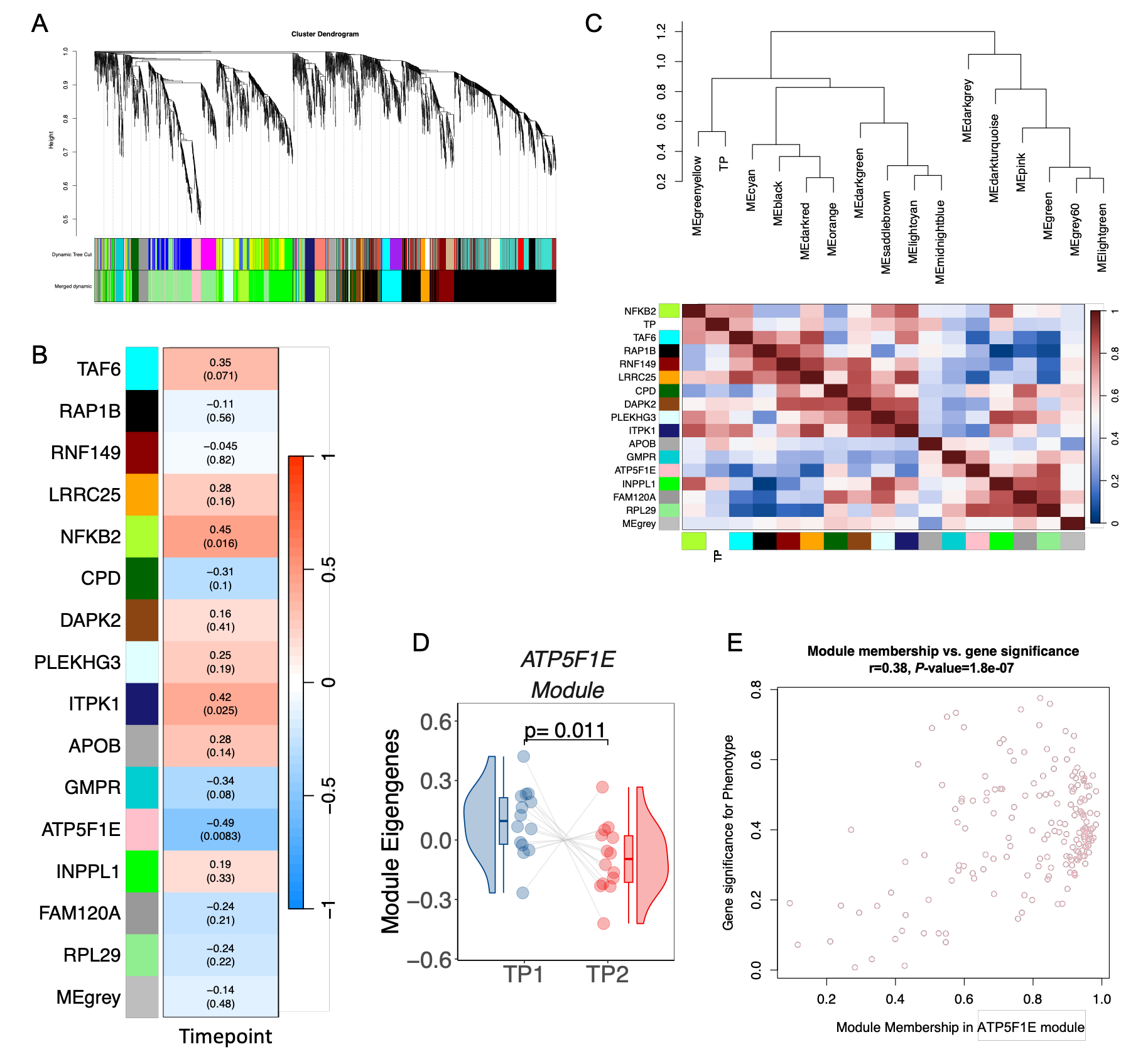


**Supplementary Note Figure 4.** (A) Top GO biological processes detected for the *ATP5F1E* and *ITPK1* modules from healthy controls. (B) Module membership (MM) and phenotype correlation (musical stimuli) for genes from the most significant pathways obtained from the module *ATP5F1E*.

**Supplementary Note Table 1.** Neuro-biological related terms retrieved from Gene Ontology database (GO).

| **GO terms** |
| --- |
| ADENYLATE CYCLASE ACTIVATING ADRENERGIC RECEPTOR SIGNALING PATHWAY |
| ADENYLATE CYCLASE ACTIVATING DOPAMINE RECEPTOR SIGNALING PATHWAY |
| ADRENAL GLAND DEVELOPMENT |
| ADRENERGIC RECEPTOR SIGNALING PATHWAY |
| AMINO ACID NEUROTRANSMITTER REUPTAKE |
| AMPA GLUTAMATE RECEPTOR CLUSTERING |
| AMYLOID BETA CLEARANCE |
| AMYLOID BETA METABOLIC PROCESS |
| AMYLOID FIBRIL FORMATION |
| AMYLOID PRECURSOR PROTEIN BIOSYNTHETIC PROCESS |
| AMYLOID PRECURSOR PROTEIN CATABOLIC PROCESS |
| AMYLOID PRECURSOR PROTEIN METABOLIC PROCESS |
| ASTROCYTE ACTIVATION |
| ASTROCYTE DEVELOPMENT |
| ASTROCYTE DIFFERENTIATION |
| AUTONOMIC NERVOUS SYSTEM DEVELOPMENT |
| AXO DENDRITIC TRANSPORT |
| AXON ENSHEATHMENT IN CENTRAL NERVOUS SYSTEM |
| BERGMANN GLIAL CELL DIFFERENTIATION |
| BRAIN MORPHOGENESIS |
| BRANCHED CHAIN AMINO ACID CATABOLIC PROCESS |
| BRANCHED CHAIN AMINO ACID METABOLIC PROCESS |
| BRANCHED CHAIN AMINO ACID TRANSPORT |
| BRANCHING MORPHOGENESIS OF A NERVE |
| BUNDLE OF HIS CELL TO PURKINJE MYOCYTE COMMUNICATION |
| CALCIUM ION REGULATED EXOCYTOSIS OF NEUROTRANSMITTER |
| CARDIAC NEURAL CREST CELL DEVELOPMENT INVOLVED IN OUTFLOW TRACT MORPHOGENESIS |
| CARDIAC NEURAL CREST CELL DIFFERENTIATION INVOLVED IN HEART DEVELOPMENT |
| CARDIAC NEURAL CREST CELL MIGRATION INVOLVED IN OUTFLOW TRACT MORPHOGENESIS |
| CATECHOLAMINE UPTAKE INVOLVED IN SYNAPTIC TRANSMISSION |
| CELL DIFFERENTIATION IN HINDBRAIN |
| CELL MIGRATION IN HINDBRAIN |
| CELL MORPHOGENESIS INVOLVED IN NEURON DIFFERENTIATION |
| CELL PROLIFERATION IN FOREBRAIN |
| CELL PROLIFERATION IN HINDBRAIN |
| CELLULAR RESPONSE TO AMYLOID BETA |
| CELLULAR RESPONSE TO CORTICOSTEROID STIMULUS |
| CELLULAR RESPONSE TO EPINEPHRINE STIMULUS |
| CELLULAR RESPONSE TO LEPTIN STIMULUS |
| CELLULAR RESPONSE TO MINERALOCORTICOID STIMULUS |
| CENTRAL NERVOUS SYSTEM DEVELOPMENT |
| CENTRAL NERVOUS SYSTEM NEURON AXONOGENESIS |
| CENTRAL NERVOUS SYSTEM NEURON DEVELOPMENT |
| CENTRAL NERVOUS SYSTEM NEURON DIFFERENTIATION |
| CENTRAL NERVOUS SYSTEM PROJECTION NEURON AXONOGENESIS |
| CERAMIDE BIOSYNTHETIC PROCESS |
| CERAMIDE CATABOLIC PROCESS |
| CERAMIDE METABOLIC PROCESS |
| CERAMIDE TRANSPORT |
| CEREBELLAR CORTEX DEVELOPMENT |
| CEREBELLAR CORTEX FORMATION |
| CEREBELLAR CORTEX MORPHOGENESIS |
| CEREBELLAR GRANULAR LAYER DEVELOPMENT |
| CEREBELLAR PURKINJE CELL LAYER DEVELOPMENT |
| CEREBELLAR PURKINJE CELL LAYER FORMATION |
| CEREBELLAR PURKINJE CELL LAYER MORPHOGENESIS |
| CEREBRAL CORTEX CELL MIGRATION |
| CEREBRAL CORTEX DEVELOPMENT |
| CEREBRAL CORTEX GABAERGIC INTERNEURON DIFFERENTIATION |
| CEREBRAL CORTEX NEURON DIFFERENTIATION |
| CEREBRAL CORTEX RADIALLY ORIENTED CELL MIGRATION |
| CEREBROSPINAL FLUID CIRCULATION |
| CIRCADIAN SLEEP WAKE CYCLE |
| CIRCADIAN SLEEP WAKE CYCLE SLEEP |
| COGNITION |
| COMMISSURAL NEURON AXON GUIDANCE |
| CORTICAL ACTIN CYTOSKELETON ORGANIZATION |
| CORTICAL CYTOSKELETON ORGANIZATION |
| CORTICOSTEROID HORMONE SECRETION |
| CORTICOSTEROID RECEPTOR SIGNALING PATHWAY |
| CRANIAL NERVE DEVELOPMENT |
| CRANIAL NERVE FORMATION |
| CRANIAL NERVE MORPHOGENESIS |
| CRANIAL NERVE STRUCTURAL ORGANIZATION |
| DENDRITE DEVELOPMENT |
| DENDRITE EXTENSION |
| DENDRITE MORPHOGENESIS |
| DENDRITE SELF AVOIDANCE |
| DENDRITIC CELL ANTIGEN PROCESSING AND PRESENTATION |
| DENDRITIC CELL APOPTOTIC PROCESS |
| DENDRITIC CELL CHEMOTAXIS |
| DENDRITIC CELL CYTOKINE PRODUCTION |
| DENDRITIC CELL DIFFERENTIATION |
| DENDRITIC CELL MIGRATION |
| DENDRITIC SPINE DEVELOPMENT |
| DENDRITIC SPINE MAINTENANCE |
| DENDRITIC SPINE MORPHOGENESIS |
| DENDRITIC TRANSPORT |
| DOPAMINE BIOSYNTHETIC PROCESS |
| DOPAMINE METABOLIC PROCESS |
| DOPAMINE RECEPTOR SIGNALING PATHWAY |
| DOPAMINE SECRETION |
| DOPAMINE TRANSPORT |
| DOPAMINERGIC NEURON DIFFERENTIATION |
| DORSAL VENTRAL NEURAL TUBE PATTERNING |
| EMBRYONIC BRAIN DEVELOPMENT |
| EMBRYONIC FORELIMB MORPHOGENESIS |
| EMBRYONIC HINDLIMB MORPHOGENESIS |
| ENSHEATHMENT OF NEURONS |
| ENTERIC NERVOUS SYSTEM DEVELOPMENT |
| EPINEPHRINE TRANSPORT |
| ESTABLISHMENT OF BLOOD BRAIN BARRIER |
| ESTABLISHMENT OF PLANAR POLARITY INVOLVED IN NEURAL TUBE CLOSURE |
| EXCITATORY SYNAPSE ASSEMBLY |
| FACIAL NERVE MORPHOGENESIS |
| FOREBRAIN CELL MIGRATION |
| FOREBRAIN DEVELOPMENT |
| FOREBRAIN GENERATION OF NEURONS |
| FOREBRAIN MORPHOGENESIS |
| FOREBRAIN NEURON DEVELOPMENT |
| FOREBRAIN NEURON DIFFERENTIATION |
| FOREBRAIN REGIONALIZATION |
| FORELIMB MORPHOGENESIS |
| G PROTEIN COUPLED ACETYLCHOLINE RECEPTOR SIGNALING PATHWAY |
| G PROTEIN COUPLED GLUTAMATE RECEPTOR SIGNALING PATHWAY |
| GABAERGIC NEURON DIFFERENTIATION |
| GAMMA AMINOBUTYRIC ACID SECRETION |
| GAMMA AMINOBUTYRIC ACID SIGNALING PATHWAY |
| GAMMA AMINOBUTYRIC ACID TRANSPORT |
| GANGLIOSIDE BIOSYNTHETIC PROCESS |
| GANGLIOSIDE METABOLIC PROCESS |
| GENERATION OF NEURONS |
| GLIAL CELL ACTIVATION |
| GLIAL CELL APOPTOTIC PROCESS |
| GLIAL CELL DERIVED NEUROTROPHIC FACTOR RECEPTOR SIGNALING PATHWAY |
| GLIAL CELL DEVELOPMENT |
| GLIAL CELL DIFFERENTIATION |
| GLIAL CELL FATE COMMITMENT |
| GLIAL CELL MIGRATION |
| GLIAL CELL PROLIFERATION |
| GLUCOCORTICOID BIOSYNTHETIC PROCESS |
| GLUCOCORTICOID METABOLIC PROCESS |
| GLUCOCORTICOID SECRETION |
| GLUTAMATE METABOLIC PROCESS |
| GLUTAMATE RECEPTOR SIGNALING PATHWAY |
| GLUTAMATE SECRETION |
| GLUTAMINE FAMILY AMINO ACID BIOSYNTHETIC PROCESS |
| GLUTAMINE FAMILY AMINO ACID CATABOLIC PROCESS |
| GLUTAMINE FAMILY AMINO ACID METABOLIC PROCESS |
| GLUTAMINE METABOLIC PROCESS |
| GLYCOSPHINGOLIPID BIOSYNTHETIC PROCESS |
| GLYCOSPHINGOLIPID METABOLIC PROCESS |
| GLYCOSYLCERAMIDE METABOLIC PROCESS |
| HINDBRAIN DEVELOPMENT |
| HINDBRAIN MORPHOGENESIS |
| HINDLIMB MORPHOGENESIS |
| HIPPOCAMPUS DEVELOPMENT |
| HISTAMINE PRODUCTION INVOLVED IN INFLAMMATORY RESPONSE |
| HISTAMINE SECRETION |
| HISTAMINE TRANSPORT |
| HYPOTHALAMUS CELL DIFFERENTIATION |
| HYPOTHALAMUS DEVELOPMENT |
| IMMUNOLOGICAL MEMORY FORMATION PROCESS |
| IMMUNOLOGICAL MEMORY PROCESS |
| IMMUNOLOGICAL SYNAPSE FORMATION |
| INHIBITORY POSTSYNAPTIC POTENTIAL |
| INHIBITORY SYNAPSE ASSEMBLY |
| INNERVATION |
| INTERNEURON MIGRATION |
| L GLUTAMATE IMPORT ACROSS PLASMA MEMBRANE |
| L GLUTAMATE TRANSMEMBRANE TRANSPORT |
| LAYER FORMATION IN CEREBRAL CORTEX |
| LEPTIN MEDIATED SIGNALING PATHWAY |
| LIMB BUD FORMATION |
| LIMBIC SYSTEM DEVELOPMENT |
| LONG TERM MEMORY |
| LONG TERM SYNAPTIC DEPRESSION |
| LONG TERM SYNAPTIC POTENTIATION |
| MAINTENANCE OF BLOOD BRAIN BARRIER |
| MAINTENANCE OF SYNAPSE STRUCTURE |
| MEMORY |
| MIDBRAIN DEVELOPMENT |
| MIDBRAIN DOPAMINERGIC NEURON DIFFERENTIATION |
| MINERALOCORTICOID BIOSYNTHETIC PROCESS |
| MINERALOCORTICOID METABOLIC PROCESS |
| MODIFICATION OF POSTSYNAPTIC STRUCTURE |
| MODIFICATION OF SYNAPTIC STRUCTURE |
| MODULATION OF EXCITATORY POSTSYNAPTIC POTENTIAL |
| MOTOR NEURON APOPTOTIC PROCESS |
| MOTOR NEURON AXON GUIDANCE |
| MOTOR NEURON MIGRATION |
| MYELIN ASSEMBLY |
| MYELIN MAINTENANCE |
| MYELOID DENDRITIC CELL ACTIVATION |
| MYELOID DENDRITIC CELL DIFFERENTIATION |
| NEGATIVE REGULATION OF AMYLOID FIBRIL FORMATION |
| NEGATIVE REGULATION OF AMYLOID PRECURSOR PROTEIN CATABOLIC PROCESS |
| NEGATIVE REGULATION OF ASTROCYTE DIFFERENTIATION |
| NEGATIVE REGULATION OF DENDRITIC SPINE DEVELOPMENT |
| NEGATIVE REGULATION OF GLIAL CELL DIFFERENTIATION |
| NEGATIVE REGULATION OF GLIAL CELL PROLIFERATION |
| NEGATIVE REGULATION OF LONG TERM SYNAPTIC POTENTIATION |
| NEGATIVE REGULATION OF MOTOR NEURON APOPTOTIC PROCESS |
| NEGATIVE REGULATION OF MYELINATION |
| NEGATIVE REGULATION OF NERVOUS SYSTEM DEVELOPMENT |
| NEGATIVE REGULATION OF NERVOUS SYSTEM PROCESS |
| NEGATIVE REGULATION OF NEURAL PRECURSOR CELL PROLIFERATION |
| NEGATIVE REGULATION OF NEUROINFLAMMATORY RESPONSE |
| NEGATIVE REGULATION OF NEURON APOPTOTIC PROCESS |
| NEGATIVE REGULATION OF NEURON DEATH |
| NEGATIVE REGULATION OF NEURON DIFFERENTIATION |
| NEGATIVE REGULATION OF NEURON MIGRATION |
| NEGATIVE REGULATION OF NEURON PROJECTION DEVELOPMENT |
| NEGATIVE REGULATION OF NEURON PROJECTION REGENERATION |
| NEGATIVE REGULATION OF NEUROTRANSMITTER SECRETION |
| NEGATIVE REGULATION OF NEUROTRANSMITTER TRANSPORT |
| NEGATIVE REGULATION OF OLIGODENDROCYTE DIFFERENTIATION |
| NEGATIVE REGULATION OF OXIDATIVE STRESS INDUCED NEURON DEATH |
| NEGATIVE REGULATION OF SYNAPSE ORGANIZATION |
| NEGATIVE REGULATION OF SYNAPTIC TRANSMISSION |
| NEGATIVE REGULATION OF SYNAPTIC TRANSMISSION GLUTAMATERGIC |
| NERVE DEVELOPMENT |
| NERVE GROWTH FACTOR SIGNALING PATHWAY |
| NERVOUS SYSTEM PROCESS |
| NERVOUS SYSTEM PROCESS INVOLVED IN REGULATION OF SYSTEMIC ARTERIAL BLOOD PRESSURE |
| NEURAL CREST CELL DIFFERENTIATION |
| NEURAL CREST FORMATION |
| NEURAL NUCLEUS DEVELOPMENT |
| NEURAL PRECURSOR CELL PROLIFERATION |
| NEURAL RETINA DEVELOPMENT |
| NEURAL TUBE DEVELOPMENT |
| NEURAL TUBE FORMATION |
| NEURAL TUBE PATTERNING |
| NEUROBLAST DIVISION |
| NEUROBLAST PROLIFERATION |
| NEUROENDOCRINE CELL DIFFERENTIATION |
| NEUROEPITHELIAL CELL DIFFERENTIATION |
| NEUROGENESIS |
| NEUROINFLAMMATORY RESPONSE |
| NEUROMUSCULAR JUNCTION DEVELOPMENT |
| NEUROMUSCULAR PROCESS |
| NEUROMUSCULAR PROCESS CONTROLLING BALANCE |
| NEUROMUSCULAR PROCESS CONTROLLING POSTURE |
| NEUROMUSCULAR SYNAPTIC TRANSMISSION |
| NEURON APOPTOTIC PROCESS |
| NEURON CELL CELL ADHESION |
| NEURON CELLULAR HOMEOSTASIS |
| NEURON DEATH |
| NEURON DEATH IN RESPONSE TO OXIDATIVE STRESS |
| NEURON DEVELOPMENT |
| NEURON FATE COMMITMENT |
| NEURON FATE DETERMINATION |
| NEURON FATE SPECIFICATION |
| NEURON MATURATION |
| NEURON MIGRATION |
| NEURON PROJECTION ARBORIZATION |
| NEURON PROJECTION EXTENSION |
| NEURON PROJECTION EXTENSION INVOLVED IN NEURON PROJECTION GUIDANCE |
| NEURON PROJECTION GUIDANCE |
| NEURON PROJECTION MAINTENANCE |
| NEURON PROJECTION ORGANIZATION |
| NEURON PROJECTION REGENERATION |
| NEURON RECOGNITION |
| NEURON REMODELING |
| NEURONAL ACTION POTENTIAL |
| NEURONAL ION CHANNEL CLUSTERING |
| NEURONAL STEM CELL POPULATION MAINTENANCE |
| NEUROPEPTIDE SIGNALING PATHWAY |
| NEUROTRANSMITTER BIOSYNTHETIC PROCESS |
| NEUROTRANSMITTER CATABOLIC PROCESS |
| NEUROTRANSMITTER GATED ION CHANNEL CLUSTERING |
| NEUROTRANSMITTER METABOLIC PROCESS |
| NEUROTRANSMITTER RECEPTOR INTERNALIZATION |
| NEUROTRANSMITTER RECEPTOR LOCALIZATION TO POSTSYNAPTIC SPECIALIZATION MEMBRANE |
| NEUROTRANSMITTER RECEPTOR TRANSPORT |
| NEUROTRANSMITTER RECEPTOR TRANSPORT ENDOSOME TO POSTSYNAPTIC MEMBRANE |
| NEUROTRANSMITTER RECEPTOR TRANSPORT TO PLASMA MEMBRANE |
| NEUROTRANSMITTER REUPTAKE |
| NEUROTRANSMITTER SECRETION |
| NEUROTRANSMITTER TRANSPORT |
| NEUROTRANSMITTER UPTAKE |
| NEUROTROPHIN SIGNALING PATHWAY |
| NEUROTROPHIN TRK RECEPTOR SIGNALING PATHWAY |
| NORADRENERGIC NEURON DIFFERENTIATION |
| NOREPINEPHRINE METABOLIC PROCESS |
| NOREPINEPHRINE SECRETION |
| NOREPINEPHRINE TRANSPORT |
| OLFACTORY BULB INTERNEURON DIFFERENTIATION |
| OLIGODENDROCYTE DEVELOPMENT |
| OLIGODENDROCYTE DIFFERENTIATION |
| OPTIC NERVE DEVELOPMENT |
| PARASYMPATHETIC NERVOUS SYSTEM DEVELOPMENT |
| PEPTIDYL GLUTAMIC ACID MODIFICATION |
| PERIPHERAL NERVOUS SYSTEM DEVELOPMENT |
| PERIPHERAL NERVOUS SYSTEM NEURON DIFFERENTIATION |
| PITUITARY GLAND DEVELOPMENT |
| PLANAR CELL POLARITY PATHWAY INVOLVED IN NEURAL TUBE CLOSURE |
| POSITIVE REGULATION OF AMYLOID BETA FORMATION |
| POSITIVE REGULATION OF AMYLOID PRECURSOR PROTEIN CATABOLIC PROCESS |
| POSITIVE REGULATION OF ASTROCYTE DIFFERENTIATION |
| POSITIVE REGULATION OF DENDRITE DEVELOPMENT |
| POSITIVE REGULATION OF DENDRITE MORPHOGENESIS |
| POSITIVE REGULATION OF DENDRITIC CELL CHEMOTAXIS |
| POSITIVE REGULATION OF DENDRITIC CELL CYTOKINE PRODUCTION |
| POSITIVE REGULATION OF DENDRITIC SPINE DEVELOPMENT |
| POSITIVE REGULATION OF DENDRITIC SPINE MORPHOGENESIS |
| POSITIVE REGULATION OF EXCITATORY POSTSYNAPTIC POTENTIAL |
| POSITIVE REGULATION OF GLIAL CELL DIFFERENTIATION |
| POSITIVE REGULATION OF GLIAL CELL MIGRATION |
| POSITIVE REGULATION OF GLIAL CELL PROLIFERATION |
| POSITIVE REGULATION OF GLUTAMATE SECRETION |
| POSITIVE REGULATION OF LONG TERM SYNAPTIC POTENTIATION |
| POSITIVE REGULATION OF MYELINATION |
| POSITIVE REGULATION OF NERVOUS SYSTEM DEVELOPMENT |
| POSITIVE REGULATION OF NERVOUS SYSTEM PROCESS |
| POSITIVE REGULATION OF NEURAL PRECURSOR CELL PROLIFERATION |
| POSITIVE REGULATION OF NEUROBLAST PROLIFERATION |
| POSITIVE REGULATION OF NEUROGENESIS |
| POSITIVE REGULATION OF NEUROINFLAMMATORY RESPONSE |
| POSITIVE REGULATION OF NEURON APOPTOTIC PROCESS |
| POSITIVE REGULATION OF NEURON DEATH |
| POSITIVE REGULATION OF NEURON DIFFERENTIATION |
| POSITIVE REGULATION OF NEURON MIGRATION |
| POSITIVE REGULATION OF NEURON PROJECTION DEVELOPMENT |
| POSITIVE REGULATION OF NEURON PROJECTION REGENERATION |
| POSITIVE REGULATION OF NEUROTRANSMITTER SECRETION |
| POSITIVE REGULATION OF NEUROTRANSMITTER TRANSPORT |
| POSITIVE REGULATION OF OLIGODENDROCYTE DIFFERENTIATION |
| POSITIVE REGULATION OF SYNAPSE ASSEMBLY |
| POSITIVE REGULATION OF SYNAPTIC TRANSMISSION |
| POSITIVE REGULATION OF SYNAPTIC TRANSMISSION GABAERGIC |
| POSITIVE REGULATION OF SYNAPTIC TRANSMISSION GLUTAMATERGIC |
| POSTSYNAPSE ASSEMBLY |
| POSTSYNAPSE ORGANIZATION |
| POSTSYNAPTIC ACTIN CYTOSKELETON ORGANIZATION |
| POSTSYNAPTIC CYTOSKELETON ORGANIZATION |
| POSTSYNAPTIC MEMBRANE ASSEMBLY |
| POSTSYNAPTIC MEMBRANE ORGANIZATION |
| POSTSYNAPTIC MODULATION OF CHEMICAL SYNAPTIC TRANSMISSION |
| POSTSYNAPTIC NEUROTRANSMITTER RECEPTOR INTERNALIZATION |
| POSTSYNAPTIC SIGNAL TRANSDUCTION |
| POSTSYNAPTIC SPECIALIZATION ASSEMBLY |
| POSTSYNAPTIC SPECIALIZATION ORGANIZATION |
| PRESYNAPSE ORGANIZATION |
| PRESYNAPTIC ENDOCYTOSIS |
| PRESYNAPTIC MODULATION OF CHEMICAL SYNAPTIC TRANSMISSION |
| PROTEIN LOCALIZATION TO CELL CORTEX |
| PROTEIN LOCALIZATION TO POSTSYNAPSE |
| PROTEIN LOCALIZATION TO PRESYNAPSE |
| PROTEIN LOCALIZATION TO SYNAPSE |
| PROTEIN POLYGLUTAMYLATION |
| PYRAMIDAL NEURON DEVELOPMENT |
| PYRAMIDAL NEURON DIFFERENTIATION |
| RADIAL GLIAL CELL DIFFERENTIATION |
| RECEPTOR LOCALIZATION TO SYNAPSE |
| REGULATION OF AMYLOID BETA CLEARANCE |
| REGULATION OF AMYLOID FIBRIL FORMATION |
| REGULATION OF AMYLOID PRECURSOR PROTEIN CATABOLIC PROCESS |
| REGULATION OF ASPARTIC TYPE ENDOPEPTIDASE ACTIVITY INVOLVED IN AMYLOID PRECURSOR PROTEIN CATABOLIC PROCESS |
| REGULATION OF ASTROCYTE DIFFERENTIATION |
| REGULATION OF CEREBELLAR GRANULE CELL PRECURSOR PROLIFERATION |
| REGULATION OF CIRCADIAN SLEEP WAKE CYCLE |
| REGULATION OF CORTICOSTEROID HORMONE SECRETION |
| REGULATION OF DENDRITE DEVELOPMENT |
| REGULATION OF DENDRITE EXTENSION |
| REGULATION OF DENDRITE MORPHOGENESIS |
| REGULATION OF DENDRITIC CELL ANTIGEN PROCESSING AND PRESENTATION |
| REGULATION OF DENDRITIC CELL CHEMOTAXIS |
| REGULATION OF DENDRITIC CELL DIFFERENTIATION |
| REGULATION OF DENDRITIC SPINE DEVELOPMENT |
| REGULATION OF DENDRITIC SPINE MAINTENANCE |
| REGULATION OF DENDRITIC SPINE MORPHOGENESIS |
| REGULATION OF DOPAMINE RECEPTOR SIGNALING PATHWAY |
| REGULATION OF DOPAMINERGIC NEURON DIFFERENTIATION |
| REGULATION OF EXCITATORY SYNAPSE ASSEMBLY |
| REGULATION OF GLIAL CELL APOPTOTIC PROCESS |
| REGULATION OF GLIAL CELL DIFFERENTIATION |
| REGULATION OF GLIAL CELL MIGRATION |
| REGULATION OF GLIAL CELL PROLIFERATION |
| REGULATION OF GLUCOCORTICOID METABOLIC PROCESS |
| REGULATION OF GLUCOCORTICOID SECRETION |
| REGULATION OF GLUTAMATE SECRETION |
| REGULATION OF LONG TERM NEURONAL SYNAPTIC PLASTICITY |
| REGULATION OF LONG TERM SYNAPTIC DEPRESSION |
| REGULATION OF LONG TERM SYNAPTIC POTENTIATION |
| REGULATION OF MICROGLIAL CELL ACTIVATION |
| REGULATION OF MOTOR NEURON APOPTOTIC PROCESS |
| REGULATION OF MYELINATION |
| REGULATION OF NERVOUS SYSTEM DEVELOPMENT |
| REGULATION OF NERVOUS SYSTEM PROCESS |
| REGULATION OF NEURAL PRECURSOR CELL PROLIFERATION |
| REGULATION OF NEUROBLAST PROLIFERATION |
| REGULATION OF NEUROGENESIS |
| REGULATION OF NEUROINFLAMMATORY RESPONSE |
| REGULATION OF NEURON DIFFERENTIATION |
| REGULATION OF NEURON MIGRATION |
| REGULATION OF NEURON PROJECTION ARBORIZATION |
| REGULATION OF NEURON PROJECTION DEVELOPMENT |
| REGULATION OF NEURON PROJECTION REGENERATION |
| REGULATION OF NEURONAL SYNAPTIC PLASTICITY |
| REGULATION OF NEUROTRANSMITTER LEVELS |
| REGULATION OF NEUROTRANSMITTER RECEPTOR ACTIVITY |
| REGULATION OF NEUROTRANSMITTER TRANSPORT |
| REGULATION OF NEUROTRANSMITTER UPTAKE |
| REGULATION OF NEUROTROPHIN TRK RECEPTOR SIGNALING PATHWAY |
| REGULATION OF OLIGODENDROCYTE DIFFERENTIATION |
| REGULATION OF POSTSYNAPSE ORGANIZATION |
| REGULATION OF POSTSYNAPTIC CYTOSOLIC CALCIUM ION CONCENTRATION |
| REGULATION OF POSTSYNAPTIC DENSITY ASSEMBLY |
| REGULATION OF POSTSYNAPTIC DENSITY ORGANIZATION |
| REGULATION OF POSTSYNAPTIC MEMBRANE NEUROTRANSMITTER RECEPTOR LEVELS |
| REGULATION OF POSTSYNAPTIC MEMBRANE POTENTIAL |
| REGULATION OF POSTSYNAPTIC NEUROTRANSMITTER RECEPTOR ACTIVITY |
| REGULATION OF POSTSYNAPTIC NEUROTRANSMITTER RECEPTOR INTERNALIZATION |
| REGULATION OF POSTSYNAPTIC SPECIALIZATION ASSEMBLY |
| REGULATION OF PRESYNAPSE ORGANIZATION |
| REGULATION OF PRESYNAPTIC CYTOSOLIC CALCIUM ION CONCENTRATION |
| REGULATION OF PROTEIN LOCALIZATION TO SYNAPSE |
| REGULATION OF RECEPTOR LOCALIZATION TO SYNAPSE |
| REGULATION OF RESPIRATORY GASEOUS EXCHANGE BY NERVOUS SYSTEM PROCESS |
| REGULATION OF SHORT TERM NEURONAL SYNAPTIC PLASTICITY |
| REGULATION OF SMOOTHENED SIGNALING PATHWAY INVOLVED IN DORSAL VENTRAL NEURAL TUBE PATTERNING |
| REGULATION OF SPHINGOLIPID BIOSYNTHETIC PROCESS |
| REGULATION OF SYNAPSE ASSEMBLY |
| REGULATION OF SYNAPSE MATURATION |
| REGULATION OF SYNAPSE STRUCTURE OR ACTIVITY |
| REGULATION OF SYNAPTIC PLASTICITY |
| REGULATION OF SYNAPTIC TRANSMISSION GABAERGIC |
| REGULATION OF SYNAPTIC TRANSMISSION GLUTAMATERGIC |
| REGULATION OF SYNAPTIC VESICLE ENDOCYTOSIS |
| REGULATION OF SYNAPTIC VESICLE EXOCYTOSIS |
| REGULATION OF SYNAPTIC VESICLE RECYCLING |
| REGULATION OF TRANS SYNAPTIC SIGNALING |
| REGULATION OF TRANSMISSION OF NERVE IMPULSE |
| RESPONSE TO ACETYLCHOLINE |
| RESPONSE TO AMYLOID BETA |
| RESPONSE TO CORTICOSTEROID |
| RESPONSE TO CORTICOSTERONE |
| RESPONSE TO DOPAMINE |
| RESPONSE TO EPINEPHRINE |
| RESPONSE TO HISTAMINE |
| RESPONSE TO L GLUTAMATE |
| RESPONSE TO LEPTIN |
| RESPONSE TO MINERALOCORTICOID |
| RESPONSE TO NERVE GROWTH FACTOR |
| ROSTROCAUDAL NEURAL TUBE PATTERNING |
| SEMAPHORIN PLEXIN SIGNALING PATHWAY INVOLVED IN NEURON PROJECTION GUIDANCE |
| SEROTONIN METABOLIC PROCESS |
| SEROTONIN RECEPTOR SIGNALING PATHWAY |
| SEROTONIN TRANSPORT |
| SEROTONIN UPTAKE |
| SHORT TERM MEMORY |
| SKELETAL MUSCLE ACETYLCHOLINE GATED CHANNEL CLUSTERING |
| SLEEP |
| SMOOTHENED SIGNALING PATHWAY INVOLVED IN DORSAL VENTRAL NEURAL TUBE PATTERNING |
| SPHINGOLIPID BIOSYNTHETIC PROCESS |
| SPHINGOLIPID MEDIATED SIGNALING PATHWAY |
| SPHINGOLIPID METABOLIC PROCESS |
| SPHINGOMYELIN BIOSYNTHETIC PROCESS |
| SPHINGOMYELIN METABOLIC PROCESS |
| SPINAL CORD ASSOCIATION NEURON DIFFERENTIATION |
| SPINAL CORD MOTOR NEURON DIFFERENTIATION |
| SPONTANEOUS SYNAPTIC TRANSMISSION |
| SYMPATHETIC NERVOUS SYSTEM DEVELOPMENT |
| SYNAPSE ASSEMBLY |
| SYNAPSE MATURATION |
| SYNAPSE ORGANIZATION |
| SYNAPSE PRUNING |
| SYNAPTIC MEMBRANE ADHESION |
| SYNAPTIC SIGNALING |
| SYNAPTIC TRANSMISSION CHOLINERGIC |
| SYNAPTIC TRANSMISSION DOPAMINERGIC |
| SYNAPTIC TRANSMISSION GABAERGIC |
| SYNAPTIC TRANSMISSION GLUTAMATERGIC |
| SYNAPTIC VESICLE CLUSTERING |
| SYNAPTIC VESICLE CYTOSKELETAL TRANSPORT |
| SYNAPTIC VESICLE DOCKING |
| SYNAPTIC VESICLE EXOCYTOSIS |
| SYNAPTIC VESICLE LOCALIZATION |
| SYNAPTIC VESICLE MATURATION |
| SYNAPTIC VESICLE MEMBRANE ORGANIZATION |
| SYNAPTIC VESICLE PRIMING |
| SYNAPTIC VESICLE RECYCLING |
| SYNAPTIC VESICLE TRANSPORT |
| SYNAPTONEMAL COMPLEX ORGANIZATION |
| TELENCEPHALON GLIAL CELL MIGRATION |
| TRANS SYNAPTIC SIGNALING MODULATING SYNAPTIC TRANSMISSION |
| TRANSMISSION OF NERVE IMPULSE |
| TRIGEMINAL NERVE DEVELOPMENT |
| VENTRAL SPINAL CORD INTERNEURON DIFFERENTIATION |
| VESICLE MEDIATED TRANSPORT IN SYNAPSE |
| VESTIBULOCOCHLEAR NERVE DEVELOPMENT |
| WNT SIGNALING PATHWAY INVOLVED IN MIDBRAIN DOPAMINERGIC NEURON DIFFERENTIATION |

**Supplementary Note Table 2.** Music-related genes from Navarro et al. [9] that are DE in TP2 vs. TP1 in ACD patients. All these genes are protein coding.

| **IDs** | **baseMean** | **log_2_FC** | **log_2_FC–SD** | **stat** | ***P*-value** | **FDR-value** | **Gene symbol** |
| --- | --- | --- | --- | --- | --- | --- | --- |
| ENSG00000002834 | 6219.8 | -0.2841 | 0.0558 | -5.0958 | 0.0000 | 0.0017 | *PABPC1* |
| ENSG00000004939 | 653.7 | 0.3496 | 0.0920 | 3.7979 | 0.0001 | 0.0164 | *ADRB2* |
| ENSG00000028116 | 334.4 | 0.2296 | 0.0624 | 3.6768 | 0.0002 | 0.0204 | *GZMK* |
| ENSG00000050130 | 670.6 | 0.3109 | 0.0866 | 3.5879 | 0.0003 | 0.0250 | *CASP3* |
| ENSG00000064012 | 787.1 | -0.3821 | 0.1074 | -3.5570 | 0.0004 | 0.0269 | *APOBR* |
| ENSG00000065978 | 738.7 | 0.2808 | 0.0830 | 3.3855 | 0.0007 | 0.0354 | *JKAMP* |
| ENSG00000068024 | 2627.1 | -0.1792 | 0.0532 | -3.3690 | 0.0008 | 0.0358 | *RNF213* |
| ENSG00000070756 | 762.2 | 0.1678 | 0.0527 | 3.1842 | 0.0015 | 0.0464 | *NPTN* |
| ENSG00000076944 | 365.0 | -0.2248 | 0.0708 | -3.1756 | 0.0015 | 0.0469 | *HDAC4* |
| ENSG00000088832 | 529.6 | 0.1910 | 0.0611 | 3.1251 | 0.0018 | 0.0496 | *TFG* |
| ENSG00000104695 | 125.0 | -0.2339 | 0.0764 | -3.0618 | 0.0022 | 0.0539 | *KCTD9* |
| ENSG00000104756 | 336.7 | 0.3604 | 0.1187 | 3.0365 | 0.0024 | 0.0554 | *CXCL8* |
| ENSG00000109332 | 664.1 | -0.3155 | 0.1058 | -2.9828 | 0.0029 | 0.0590 | *SLC6A8* |
| ENSG00000109756 | 1909.5 | 0.2181 | 0.0734 | 2.9715 | 0.0030 | 0.0606 | *CASP8* |
| ENSG00000111859 | 8816.9 | 0.1966 | 0.0697 | 2.8210 | 0.0048 | 0.0745 | *UBE2D3* |
| ENSG00000112893 | 2768.6 | 0.1311 | 0.0472 | 2.7792 | 0.0054 | 0.0798 | *DUSP6* |
| ENSG00000113088 | 3989.2 | -0.2338 | 0.0845 | -2.7666 | 0.0057 | 0.0816 | *YBX1* |
| ENSG00000114354 | 273.0 | 0.1846 | 0.0669 | 2.7617 | 0.0058 | 0.0819 | *ZNF83* |
| ENSG00000114861 | 580.4 | 0.1924 | 0.0701 | 2.7461 | 0.0060 | 0.0839 | *HIGD1A* |
| ENSG00000114978 | 2584.2 | 0.1378 | 0.0511 | 2.6984 | 0.0070 | 0.0889 | *CX3CR1* |
| ENSG00000115310 | 1146.9 | 0.1768 | 0.0658 | 2.6859 | 0.0072 | 0.0905 | *DSTN* |
| ENSG00000115758 | 103.1 | 0.2963 | 0.1117 | 2.6532 | 0.0080 | 0.0951 | *PKIA* |
| ENSG00000118260 | 1869.3 | 0.1857 | 0.0702 | 2.6475 | 0.0081 | 0.0951 | *ODC1* |
| ENSG00000125868 | 1777.4 | 0.2082 | 0.0806 | 2.5834 | 0.0098 | 0.1046 | *TLR1* |
| ENSG00000130821 | 104.5 | 0.3192 | 0.1252 | 2.5490 | 0.0108 | 0.1101 | *HDC* |
| ENSG00000135336 | 1342.0 | -0.1367 | 0.0547 | -2.4990 | 0.0125 | 0.1198 | *RTN4* |
| ENSG00000139318 | 1690.5 | 0.1506 | 0.0606 | 2.4843 | 0.0130 | 0.1230 | *SDHD* |
| ENSG00000140287 | 6115.3 | -0.1650 | 0.0678 | -2.4322 | 0.0150 | 0.1311 | *RPS9* |
| ENSG00000142197 | 944.2 | -0.1469 | 0.0610 | -2.4081 | 0.0160 | 0.1356 | *FOXP1* |
| ENSG00000156642 | 1866.8 | 0.2115 | 0.0881 | 2.4017 | 0.0163 | 0.1366 | *FOS* |
| ENSG00000164305 | 1188.8 | 0.1605 | 0.0670 | 2.3967 | 0.0165 | 0.1367 | *CREB1* |
| ENSG00000167766 | 287.1 | 0.1708 | 0.0713 | 2.3953 | 0.0166 | 0.1367 | *ORC3* |
| ENSG00000168329 | 242.4 | -0.1750 | 0.0731 | -2.3930 | 0.0167 | 0.1368 | *RAPGEF2* |
| ENSG00000169252 | 1099.2 | -0.1610 | 0.0693 | -2.3247 | 0.0201 | 0.1491 | *LASP1* |
| ENSG00000169429 | 166.5 | -0.1725 | 0.0748 | -2.3065 | 0.0211 | 0.1524 | *DOP1B* |
| ENSG00000170345 | 964.3 | 0.1352 | 0.0595 | 2.2714 | 0.0231 | 0.1598 | *NEDD9* |
| ENSG00000170889 | 164.5 | -0.2096 | 0.0932 | -2.2490 | 0.0245 | 0.1663 | *PPP2CB* |
| ENSG00000171033 | 589.1 | -0.1444 | 0.0644 | -2.2435 | 0.0249 | 0.1674 | *MAN2A1* |
| ENSG00000173821 | 2142.1 | 0.1478 | 0.0669 | 2.2075 | 0.0273 | 0.1765 | *MYADM* |
| ENSG00000174125 | 541.4 | -0.1757 | 0.0797 | -2.2058 | 0.0274 | 0.1769 | *FKBP1A* |
| ENSG00000179820 | 157.7 | 0.1906 | 0.0877 | 2.1726 | 0.0298 | 0.1857 | *VRK2* |
| ENSG00000181061 | 3247.8 | 0.1147 | 0.0536 | 2.1397 | 0.0324 | 0.1929 | *MOB1A* |
| ENSG00000183813 | 1246.7 | -0.1112 | 0.0542 | -2.0535 | 0.0400 | 0.2148 | *STXBP2* |
| ENSG00000184678 | 454.6 | 0.1894 | 0.0934 | 2.0282 | 0.0425 | 0.2208 | *H2BC21* |
| ENSG00000184730 | 2225.6 | -0.1612 | 0.0797 | -2.0241 | 0.0430 | 0.2214 | *SLC4A1* |
| ENSG00000204370 | 146.3 | -0.2153 | 0.1073 | -2.0069 | 0.0448 | 0.2253 | *PLIN5* |
| ENSG00000211456 | 553.9 | 0.1334 | 0.0680 | 1.9620 | 0.0498 | 0.2394 | *SACM1L* |
| ENSG00000214456 | 86.4 | 0.2526 | 0.1285 | 1.9658 | 0.0493 | NA | *CCR4* |
