# Supplementary material for: Music compensates for altered gene expression in age-related cognitive disorders
Source: Sci Rep. 2023 Dec 2;13:21259. doi: 10.1038/s41598-023-48094-5 (PMC10692168; doi:10.1038/s41598-023-48094-5)
Supplement: Supplementary file 13 — Supplementary Legends. [file 41598_2023_48094_MOESM13_ESM.docx]

**Supplementary Data**

**Supplementary Table S1.** DEGs detected between TP1 and TP2 in the ACD and healthy control cohorts.

**Supplementary Table S2.** Biological processed terms differentially activated in ACD and healthy controls between TP1 and TP2, as derived from GSVA analysis; and neuro-biological related terms differentially activated between ACD patients and healthy controls in response to musical stimuli, as derived from QuSAGE analysis.

**Supplementary Table S3.** Modules of co-expressed genes detected in the AD patients and healthy controls cohorts and their correlation with the musical stimuli (FDR=false discovery rate). Over-representation analysis of ACD and healthy controls modules, using Gene Ontology (GO) database as reference.

**Supplementary Table S4.** Correlation between shared DEGs and DEPs obtained from MCI/AD *vs*. controls (multi-cohort case-control study) and from TP2 *vs*. TP1 in ACD patients (Pretest-Posttest study). Enrichment analysis results from the PPI network of the shared DEGs. In the enrichment map, nodes represent gene-sets and edges represent mutual overlap (highly redundant gene-sets are grouped together as clusters) [12].

**Supplementary Figure S1**. Expression values heatmap of all DEGs (*P*-value <0.05) between TP1 and TP2 in both ACD and healthy controls cohorts.

**Supplementary Figure S2**. GSVA showing differences detected in the activation or inhibition of the main neuro-biological pathways in patients and controls when exposed to music (FDF<0.05)

**Supplementary Figure S3**. Correlation between activation values (represented as log_2_FC) from shared significant (FDR<0.05) neuro-biological pathways detected in ACD and healthy controls cohorts.

**Supplementary Figure S4**. QuSAGE analysis between ACD patients and healthy controls. (A) QuSAGE results showing the top differentially activated pathways between ACD patients and controls related to neuro-biological processes (*P*-value<0.05; |*PA_ACD_*|+|*PA_HC_*|>0.05). (B) Activity of individual genes belonging to the pathway “negative regulation of amyloid beta clearance”.

**Supplementary Figure S5**. Co-expression network analysis in ACD patient’s cohort. A) Clustering dendrogram of genes and co-expression modules detected, represented by different colors. (B) Hierarchical clustering eigengene dendrogram and heatmap for ACD patients’ datasets showing relationships among the modules and TP (musical stimuli; TP2 *vs.* TP1). Gene names on the left of the heatmap are the hub genes of each module. (C) Plots showing comparison between MM (module membership) and musical stimuli correlation of genes from the significant (FDR<0.05) modules detected in the ACD cohort.

**Supplementary Figure S6.** PPI network and downstream enrichment analysis of the proteins coded by the top A) 207 DEGs in MCI *vs*. healthy controls, and B) 131 DEGs in AD *vs*. healthy controls, and impacted by musical stimuli in ACD patients (FDR < 0.05 in both studies).

**Supplementary Figure S7**. Correlation of log_2_FC for the DEGs and DEPs detected in MCI (when compared against healthy controls) and the DEGs and DEPs detected in AD (when compared against healthy controls) that are also significant in ACD after the musical stimuli (see **Supplementary Table S4**, **Figure 4 and Figure 5** for details). For all the correlation values, *P*-value < 2.22×10^-16^.
